# Complex alternative splicing of human Endonuclease V mRNA, but evidence for only a single protein isoform

**DOI:** 10.1371/journal.pone.0225081

**Published:** 2019-11-08

**Authors:** Natalia Berges, Meh Sameen Nawaz, Tuva Børresdatter Dahl, Lars Hagen, Magnar Bjørås, Jon K. Laerdahl, Ingrun Alseth

**Affiliations:** 1 Department of Microbiology, Oslo University Hospital Rikshospitalet and University of Oslo, Oslo, Norway; 2 Research Institute of Internal Medicine, Oslo University Hospital Rikshospitalet and Institute of Clinical Medicine, University of Oslo, Oslo, Norway; 3 Department of Clinical and Molecular Medicine, Norwegian University of Science and Technology, Trondheim, Norway; 4 PROMEC Core Facility for Proteomics and Modomics, Norwegian University of Science and Technology and Central Norway Regional Health Authority, Trondheim, Norway; Shanghai Ocean University, CHINA

## Abstract

Endonuclease V (ENDOV) is a ribonuclease with affinity for inosine which is the deamination product of adenosine. The genomes of most organisms, including human, encode ENDOV homologs, yet knowledge about *in vivo* functions and gene regulation is sparse. To contribute in this field, we analyzed mRNA and protein expression of human ENDOV (hENDOV). Analyses of public sequence databases revealed numerous *hENDOV* transcript variants suggesting extensive alternative splicing. Many of the transcripts lacked one or more exons corresponding to conserved regions of the ENDOV core domain, suggesting that these transcripts do not encode for active proteins. Three complete transcripts were found with open reading frames encoding 282, 308 and 309 amino acids, respectively. Recombinant hENDOV 308 and hENDOV 309 share the same cleavage activity as hENDOV 282 which is the variant that has been used in previous studies of hENDOV. However, hENDOV 309 binds inosine-containing RNA with stronger affinity than the other isoforms. Overexpressed GFP-fused isoforms were found in cytoplasm, nucleoli and arsenite induced stress granules in human cells as previously reported for hENDOV 282. RT-qPCR analysis of the 3’-termini showed that *hENDOV 308* and *hENDOV 309* transcripts are more abundant than *hENDOV 282* transcripts in immortalized cell lines, but not in primary cells, suggesting that cells regulate *hENDOV* mRNA expression. In spite of the presence of all three full-length transcripts, mass spectrometry analyses identified peptides corresponding to the hENDOV 309 isoform only. This result suggests that further studies of human ENDOV should rather encompass the hENDOV 309 isoform.

## Introduction

Endonuclease V (ENDOV) is a ubiquitous enzyme found in all three domains of life [1,2]. ENDOV has affinity towards inosines, which are deaminated adenosines present in both DNA and RNA [3,4]. In DNA inosine is seen asadamage [5], whereas in RNA inosine is enzymatically introduced and is one of the most abundant non-cognate bases [6]. Inosine is read as guanosine by cellular proteins, and hence, in DNA is mutagenic [7]. In RNA, consequences will depend on where in the RNA transcript the deaminations occur and include translational recoding, altered recognition sites, change of splice sites and destabilization of hairpin structures, among others [8]. In tRNA, inosines in the wobble position are essential for efficient protein translation [9].

ENDOV recognizes inosine and cleaves the second phosphodiester bond 3’ of the deaminated base, leaving 3’-OH and 5’-P termini, in a Mg^2+^/Mn^2+^ dependent manner [4]. The crystal structure of ENDOV shows a globular enzyme with a central β-sheet comprising eight β-strands flanked by seven α-helixes and contains an “RNase H-like motif” [1,10]. Originally, ENDOV was characterized as a DNA repair protein in bacteria. Recent research has shown that several eukaryotic homologs, including the human enzyme, are inactive on DNA, and rather have robust inosine-dependent ribonuclease activity [10–12]. In accordance with the RNA selectivity, ectopically expressed human ENDOV (hENDOV) has mainly a cytoplasmic subcellular localization [13] with some nucleolar staining [14]. When exposed to oxidative stress, ectopically expressed hENDOV relocalizes to cytoplasmic stress granules, suggesting that hENDOV activity is regulated upon certain stresses [13].

Whereas the *in vitro* activity of mammalian ENDOV is well studied, the *in vivo* function remains enigmatic. Bacterial mutants of ENDOV have been characterized, and except a moderate increase in mutation frequency, no distinct phenotype has been reported [15]. ENDOV from the unicellular eukaryote parasite *Trypanosoma brucei* has, similarly as hENDOV, a strong preference for inosine in RNA over DNA [16]. The enzyme appears to be non-essential in bloodstream forms of the parasite, that is, the mammalian stage. In contrast, protein depletion in the procyclic forms (insect-stage parasites) leads to impaired growth and defects in cell cycle progression, suggesting a specific role for *T*. *brucei* ENDOV at this life stage [15]. To our knowledge, no reports on mammalian organisms lacking ENDOV have been published,. However, as shown in [13], ENDOV depleted human cells are viable with normal growth.

Splicing of precursor messenger RNAs (pre-mRNAs) is a critical step in post-transcriptional gene regulation, providing diversity of the functional proteome of eukaryotic organisms. The average human gene contains eight exons and seven introns, producing an average of three or more alternatively spliced mRNA isoforms [17]. Alternative mRNA splicing results in protein isoforms that may have separate biological properties that could play important roles in various biological processes, including pathologies [18]. It is therefore important to understand regulation, function and amounts of different isoforms. For *hENDOV*, public databases show a complex expression and splicing pattern [14]. In general, *hENDOV* mRNA levels are low in most tissues and alternative splicing is comprehensive. Moreover, which of these transcripts are translated into functional proteins in human cells is unclear. To address this, we have carefully analyzed and summarized transcript data available in public databases and performed thorough analyses of *hENDOV* transcripts and protein isoforms in human cells and cell lines. Interestingly, we find no evidence for the “canonical” and commonly used hENDOV 282 protein isoform. Rather hENDOV exists in the cells with an alternative C-terminus not previously described.

## Material and methods

### Sequence analyses

*hENDOV* transcript and protein sequence analysis was performed with data from the NCBI Entrez Gene [19] and RefSeq [20] database resources, and from the Ensembl [21] and UniProt [22] databases. Protein subcellular localization predictions were performed with WoLF PSORT (https://wolfpsort.hgc.jp/) and protein structural disorder predictions with DISOPRED3 [23].

### Ethics

The protocols were approved by the Regional Committee for Medical and Research Ethics in South‐East Norway, REK no S-06172. All participants gave signed informed consent and the study protocols were in agreement with the principles of the Declaration of Helsinki.

### Cell culture

The human testicular germ-cell tumor cell line 833K [24,25] was a generous gift from Dr. Anne Karin Olsen (Norwegian Institute of Public Health, Oslo, Norway). These cells were grown in RPMI 1640 media (Thermo Fisher Scientific, 21875) with 10% fetal bovine serum (FBS; Thermo Fisher Scientific, 10099141) and 1% penicillin-streptomycin (P/S; Thermo Fisher Scientific, 15140122). HAP1 (derived from male chronic myelogenous leukemia cell line KBM-7) cell line (Horizon Discovery, wild type C631, *ENDOV*^-^ HZGHC002792c012) was grown in IMDM media (Merck, I6529) with 10% FBS (Merck, F7524) and 1% P/S. The hepatocyte derived cellular carcinoma cell line Huh-7 (Health Science Research Resources Bank, JCRB0403) was grown in DMEM, low glucose media (Thermo Fisher Scientific, 22320) with 10% FBS (Merck, F7524) and 1% P/S. The human embryonic kidney cell line HEK 293T (American type Culture Collection (ATCC), CRL-11268) was grown in DMEM, high glucose, GlutaMAX(TM) media (Thermo Fisher Scientific, 10566) with 10% FBS (Merck, F7524) and 1% P/S. The human monocytic cell line THP-1 (ATCC, TIB-202) was grown in RPMI 1640 media (Biowest, L0496–500) supplemented with 10% FBS (Merck, F7524) and 1% P/S (Merck, P4458). All cells were kept at 37 °C and 5% CO_2_.

### Polarization of THP-1 cells

Cells were seeded in 24-well plates in growth media and stimulated with phorbol 12-myristate 13-acetate (PMA, 100 nM; Merck, P8139) for 6 hours (h) at 37 °C for macrophage differentiation. Following differentiation, cells were treated with growth media containing lipopolysaccharide (LPS, 10 ng/mL, Merck, L4391) and interferon γ (INFγ, 5 ng/mL, R&D Systems, 285-IF) or interleukin 4 (IL-4, 25ng/ml R&D Systems, 204-IL) and IL-13 (25 ng/mL, R&D Systems, 213-ILB) for 24 h for M1 or M2 polarization, respectively. The specific macrophage polarization was validated by measuring the mRNA expression of M1 and M2 markers in the cells by real-time quantitative PCR (RT-qPCR). Two independent polarization experiments were performed.

### Isolation and polarization of human primary monocytes

Human peripheral blood mononuclear cells (hPBMCs) were isolated from buffy coats of healthy donors (Blodbanken i Oslo, Oslo Universitetessykehus) by Isopaque-Ficoll (Lymphoprep; Alere, A80102010) gradient centrifugation and incubated in flasks with 10 mL serum-free RPMI 1640 media (Biowest, L0496–500) for 90 minutes (min) for plastic adherence of monocytes before washing. Cells were then incubated for 6 days with RPMI 1640 media 10% FBS (Merck, F7524) and), 1% P/S (Merck, P4458) and 20 ng/mL macrophage colony stimulating factor (M-CSF; R&D Systems, 216-MC). For polarization, the cells were seeded in a 24-well plate and polarization was obtained towards the M1 or M2 phenotypes by incubation with RPMI 1640 supplemented with LPS (100 ng/mL) and IFNγ (20 ng/mL) for M1 polarization or IL-4 (20 ng/mL) for M2 polarization, for 18 h. The specific macrophage polarization was validated by measuring the mRNA expression of M1 and M2 markers in the cells by RT-qPCR. Cells isolated from a buffy coat were used in only one independent experiment, with multiple biological replicates. Two independent isolations from different buffy coats were performed.

### RNA extraction and RT-qPCR

RNA was isolated from each cell line using miRNeasy mini kit (Qiagen, 217004). cDNA was generated using Quantitect Reverse Transcription Kit (Qiagen, 205313). RT-qPCR was performed using Step One Plus Real-time PCR system (Applied Biosystem) and PowerUp SYBR Green PCR master mix (Thermo Fisher Scientific, A25742) according to the system instructions. In each reaction 25 ng cDNA was used for the immortalized cell lines and 5 ng for the immune cell line THP-1 and the primary cells. The experiments were repeated twice and samples run in parallels. *Hypoxanthine phosphoribosyltransferase 1* (*HPRT1*), *glyceraldehyde-3-phosphate dehydrogenase* (*GAPDH*) and *beta-actin* (*ACTB*) were used as reference genes for normalization. Melting point analyses were performed to confirm the specificity of the PCR products. Raw data was analyzed using LinReg PCR (version 2016.1, download: https://www.medischebiologie.nl/files/) and all samples were related to the average of *hENDOV 282* samples, as indicated in the figure texts. Primer sequences used for these analyses are available on request.

### PCR

Briefly, RNA was isolated and cDNA synthesized as described above. For each reaction, 250 ng cDNA was used and positive controls in the PCR were corresponding GFP-expression plasmids for *hENDOV 282* and *hENDOV 309*. For *hENDOV 308* the GFP-expression plasmid could not be used due to primer mismatch because of codon optimization. GoTaq^®^ Green Master Mix (Promega, M712) was used and PCR products were separated on a 1.5% agarose gel, DNA excised and analyzed by DNA sequencing. Primer sequences are available on request.

### Purification of recombinant hENDOV proteins

The *hENDOV* 282, *hENDOV* 308, *hENDOV* 309, hENDOV C-terminal deletion variant (amino acids 1–250; hENDOV 250), a variant lacking exon 3 (hENDOV 282-E3) and a variant with mutated glutamate E171 and arginine R174 to alanine (AA) in the α4-helix (hENDOV 282AA) open reading frames were cloned into vector pETM41 (Genescript; [14]) and expressed with an N-terminal His-MBP tag. Protein purifications were performed as previously described [13].

### Activity and electrophoretic mobility shift assays

Activity measurements were done as previously described [13] using ^32^P 5’-labelled RNA substrates (single-stranded (ss) IIUI 5’-ACUGGACA[rI][rI]U[rI]CUCCGAGG or double-stranded (ds) IIUI (ssIIUI annealed to complementary CCUCGGAGU[rI]UUUGUCCAGU)), enzyme amounts as indicated, in 10 mM Tris-HCl pH 7.5, 0.5 mM MnCl_2_, 50 mM KCl, 1 mM DTT, 5% glycerol. After separation, the gels were dried and radiolabelled fragments visualized by phosphorimaging (Typhoon 9410 Variable Mode Imager). ImageQuant TL was used for quantification. All experiments were performed at least three times and representative experiments are shown. The graphs show the average of three independent experiments with SEM.

Bandshift assays were performed using the same RNA substrates as above in 70 mM morpholinopropanesulfonic acid (MOPS pH 7.5), 1 mM DTT, 1 mM EDTA, 50 mM CaCl_2_ and 5% glycerol with enzyme amounts as indicated. As competitor, 1 ng/μL tRNA from *Escherichia coli* (*E*. *coli)* (Merck, R1753) was added. Reactions were incubated on ice for 15 min and added loading buffer. hENDOV-substrate complexes were separated from unbound substrates by running on 12.5% native polyacrylamide gels with 5% glycerol in 0.5x taurine at 150 V for 45 min on ice. After separation, the gels were dried and radiolabelled fragments visualized by phosphorimaging and quantified as above. All experiments were performed at least three times and representative experiments are shown. The quantification shows the average of three independent experiments with SEM.

### Whole cell lysates and subcellular fractionation

Whole cell extracts from HAP1 cells were prepared by harvesting cells in lysis buffer containing 20 mM Tris-HCl pH 8.0, 100 mM NaCl, 0.5% IGEPAL^®^ CA-630 (Merck, 9002-93-1) and freshly added protease inhibitor cocktail (PIC, 1:100, Merck, P8340), 1 mM DTT and 400 μM ribonucleoside vanadyl complex (VRC, BioNordika/NEB, S1402S). After 30 min of incubation on ice, extracts were cleared by centrifugation at 14,000 g for 15 min at 4°C.

Nuclear and cytoplasmic fractions from HAP1 and HEK 293T cells ectopically expressing GFP fused hENDOV isoforms were prepared by lysing cells in cytoplasmic extraction buffer containing 10 mM 4-(2-hydroxyethyl)-1-piperazineethanesulfonic acid (HEPES; pH 7.5), 10 mM KCl, 0.1 mM EDTA, 1 mM DTT, 0.5% IGEPAL^®^ CA-630 and 0.5 mM PMSF (Merck, P7626) added freshly along with PIC (1:100). After 30 min of intermittent mixing on ice, extracts were centrifuged at 14,000 g for 10 min at 4 °C. The supernatant was further used as cytoplasmic fraction. The pelleted nuclei were washed three times with cytoplasmic extraction buffer and resuspended in the nuclear extraction buffer containing 20 mM HEPES pH 7.5, 400 mM NaCl, 1 mM EDTA, 1 mM DTT and freshly added 1 mM PMSF and PIC (1:100). The nuclei were sonicated and incubated on ice for 30 min, followed by centrifugation at 14,000 g for 5 min at 4 °C. The cleared extracts were used as nuclear fractions. Protein concentration was measured using Bradford assay with Protein Assay Dye Reagent Concentrate (Bio-Rad, 5000006). To verify the efficiency of subcellular fractionation, up to 20 μg of nuclear, cytoplasmic and whole cell extracts were subjected to western blot analysis. 2x Laemmli buffer (Bio-Rad, 1610747) was used to prepare the samples. After denaturation at 95 °C for 5 min, proteins were separated by PAGE (12%, Mini-PROTEAN, Bio-Rad, 4561043), transferred to PVDF membranes (Bio-Rad, 1704156), and the membranes were blocked with PBS with 0.05% Tween 20 (PBS-T; Merck, P1379) and 5% skimmed milk (Merck, 70166) for 1 h at room temperature. Precision Plus Protein^™^ Dual Color Standards (Bio-rad, 1610374) was used as molecular weight marker. α-Tubulin and Histone H3 (used as cytoplasmic and nuclear markers, respectively) were detected using primary antibodies anti-α-Tubulin (1:3000; Merck, T5168) and anti-Histone H3 (1:3000; Abcam, ab1791) in PBS-T with 5% skimmed milk for 1 h at room temperature. Anti-GFP antibody (1:1000; Abcam, ab290) and anti-hENDOV antibody (1:100; in-house monoclonal [12]) were used to detect GFP fused hENDOV isoforms. The membranes were washed three times for 10 min with PBS-T prior and post-incubation with secondary antibodies ECL Rabbit IgG horseradish peroxidase (HRP)-linked whole antibody (1:10000, GE Healthcare, NA934) or ECL Mouse IgG, HRP-linked whole antibody (1:10000, GE Healthcare, NA931) for 1 h. Substrate SuperSignal^™^ West Femto (Thermo Fisher Scientific, 34095) was used to detect the signals and the membranes were analyzed with Image Lab^™^ version 5.1 software from Bio-Rad.

### IP of endogenous and recombinant hENDOV

Endogenous hENDOV was immunoprecipitated from protein extracts from different cell lines using a commercially available ENDOV antibody (Abcam, ab69400). Whole cell protein extracts (2–3 mg); nuclear (1.5 mg) and cytoplasmic (1.5 mg) fractions were incubated with 1.5 μg antibody in 1 mL of lysis buffer. A sample with 70 ng recombinant hENDOV 282 was included as positive control. The mixtures were incubated for 4 h with slow rotation at 4 °C. 20 μL Protein A/G Plus-agarose beads (Santa Cruz, sc-2003) were added and incubation continued overnight. The beads were collected by centrifugation at 600 g for 5 min at 4 °C and washed three times with washing buffer (20 mM Tris-HCl pH 8.0, 150 mM NaCl and 0.5% IGEPAL^®^ CA-630 with freshly added PIC (1:100), 1 mM DTT and 400 μM VRC). After the last wash, the liquid was removed. Samples analyzed by mass spectrometry (MS) were resuspended in 250 μL PBS and PIC (1:100). For endonuclease activity, samples were added 1 μL of ^32^P 5’-labelled ssIIUI RNA substrate (described above), 1 mM DTT, 40 U RNAseOUT (Life, 10777019) and reaction buffer (described above) to a total volume of 15 μL. Reactions proceeded at 37 °C for 15 min and were stopped by adding 20 μL formamide loading dye (80% formamide, 10 mM EDTA, 0.1% xylene cyanol and bromophenol blue). The samples were heated at 50 °C for 5 min and centrifuged for 3 min at 8 g prior to separation on gels and visualization as described above. Recombinant hENDOV isoforms (0.15 μg) were immunoprecipitated prior to western analysis as described above, to verify antibody recognition of all isoforms used. An in-house polyclonal hENDOV antibody (1:1000) [12] was used to detect hENDOV isoforms.

### Targeted mass spectrometry analysis

Magnetic MagReSyn^®^ HILIC beads (ReSyn biosciences, MR-HLC002) were equilibrated in 15% Pierce^™^ Acetonitrile (ACN), 100 mM ammonium acetate (NH_4_Ac, pH 4.5), removed from the liquid and further incubated with immunoprecipitated hENDOV mixed 1:1 with 30% ACN, 200 mM NH_4_Ac (pH 4.5). Beads were washed twice with 95% ACN and finally digested overnight using 0.5 μg/μL trypsin in 50 mM NH_4_HCO_3_. Peptides were acidified to 0.5% acetic acid and desalted using the StageTip protocol [26] before MS-analysis.

Purified recombinant hENDOV proteins (hENDOV 282, hENDOV 308 and hENDOV 309) were used to generate peptide standards for targeted MS analysis. Common peptides for hENDOV (exon 2) and peptides specific for the different C-termini were used.

Parallel reaction monitoring (PRM)-based targeted MS methods were performed as previously described [27] with minor modifications. Peptides were analyzed in a Q Exactive HF mass spectrometer coupled to an Easy-nLC 1200 UHPLC system (Thermo Fisher Scientific). Peptides were injected onto an Acclaim PepMap100 C-18 column and further separated on an Acclaim PepMap100 C-18 analytical column (Thermo Fisher Scientific). A 120 min method was used at a 250 nL/min flow rate: starting with 95% buffer A (0.1% formic acid) with an increase to 40% buffer B (80% acetonitrile, 0.1% formic acid) in 75 min, followed by an increase to 100% B over 5 min, where it was subsequently held for 10 min. Peptides eluting from the column were analyzed on the Q Exactive operating in PRM mode. Experimental design, data analysis and processing were performed using the Skyline software (version 4.1.0.18169).

Peptides generated after digestion of recombinant hENDOV proteins were first analyzed in a Q Exactive HF and the data imported into Skyline where the top 2–8 ionizing peptides (2+ or 3+ charge states) were selected for PRM analysis. Here, information on retention time and fragmentation pattern of the standards was used to identify the peptides for each protein and to build a scheduled PRM method with a retention time window of 8 min. The method was then used for detection and quantification of corresponding peptides.

### Northern blot analyses

Total RNA from HAP1 cells (isolated as described above) was incubated with 75 or 150 pmol recombinant hENDOV as described above for the activity assays. A sample with only RNA (no enzyme) and another sample with 150 pmol BSA (Bionordika/NEB, B9000S) were included as controls. Equal volume of formamide loading dye was added and the samples were heated at 50°C for 5 min before separation by 15% denaturating PAGE (7 M urea and 1x taurine) at 200 V for 50 min in 1x taurine. One of the gels was stained with ethidium bromide to check for RNA integrity. The RNA was transferred to a nylon membrane (Hybond XL, GE Healthcare, RPN203S) by electroblotting in 1x taurine at 5 V for 1 h at room temperature. RNA was UV-crosslinked to the membranes (120 mJ/cm-2 in a CL-1000 UV-Crosslinker, UVP). The Northern Max kit (Thermo Fisher Scientific, AM1940) was used for prehybridization, hybridization and washing steps as described by the manufacturer. ^32^P 5’-labelled oligonucleotides (Eurofins) complementary to the tRNAs: AlaAGC5’, ArgACG5’ and ValAAC5’ were used as probes (sequences available on request). Hybridization signals were analyzed by phosphorimaging and quantified by ImageQuant TL software using background substraction rolling ball. Hybridized probes were removed from the filters by boiling in 0.1% SDS.

### Subcellular localization of ectopically expressed hENDOV isoforms

The following hENDOV full-length and truncated versions fused to the C-terminus of green fluorescent protein (GFP) were made for localization analyses: hENDOV 282, hENDOV 308, hENDOV 309, hENDOV 250, hENDOV 282-E3 and hENDOV 282AA (Genescript; [14]). HEK 293T cells were transiently transfected with constructs expressing the different GFP-hENDOV isoforms using FuGENE 6 transfection reagent (Promega, E2691) according to the manufacturer’s protocol. Cells were grown on 12 mm coverslips coated with fibronectin (20 μg/mL, Merck, F1141) until 70–80% confluence was reached. Arsenite-treated cells were exposed to 0.5 mM sodium arsenite (Merck, 35000) for 30 min for stress granule formation. Cells were washed in PBS before fixation in 4% paraformaldehyde in PBS for 15 min. After washing in PBS twice, cells were quenched in PBS with 0.1 M glycine (Merck, G8898) for 10 min, permeabilized in PBS with 0.1% Triton X100 (Merck, T8787) for 10 min and incubated in blocking buffer (PBS with 0.5% BSA; Saveen Werner, B2000–500) for 30 min. All subsequent labelling steps were performed in blocking buffer and cells were incubated for 1 h with primary and secondary antibodies. Antibodies against G3BP (1:100; BD Biosciences, 611126 or 1:500; Abcam, ab181150) were used to detect stress granules. Fibrillarin was used as a nucleolar marker (1:500; Abcam, ab4566). Alexa 594- (1:1000; Life, A11012) or 647- (1:1000; Life, A21235) conjugated anti-rabbit or anti-mouse IgG antibodies were used for detection. Nuclei were stained with DAPI (Thermo Fisher Scientific, D1306) and coverslips were mounted with Mowiol (Merck, 475904). Confocal images were acquired with Leica TCS SP8 equipped with a 40x/1.3 NA oil immersion objective using 6x-10x zoom.

### Statistical analyses

Comparison of the mean between more than two groups was conducted using one-way ANOVA followed by Tukey’s multiple comparisons test or two-way ANOVA followed by Tukey’s multiple comparisons test. The software GraphPad Prism (version 7.03 for Windows, GraphPad Software, La Jolla California USA, www.graphpad.com/) was used for the analyses. Differences were considered statistically significant at p < 0.05 (*p < 0.05; **p < 0.01; ***p < 0.001). Data are presented as mean ± standard error of mean (SEM) unless otherwise stated.

## Results

### Analysis of annotated *hENDOV* isoforms

**Fig 1:**
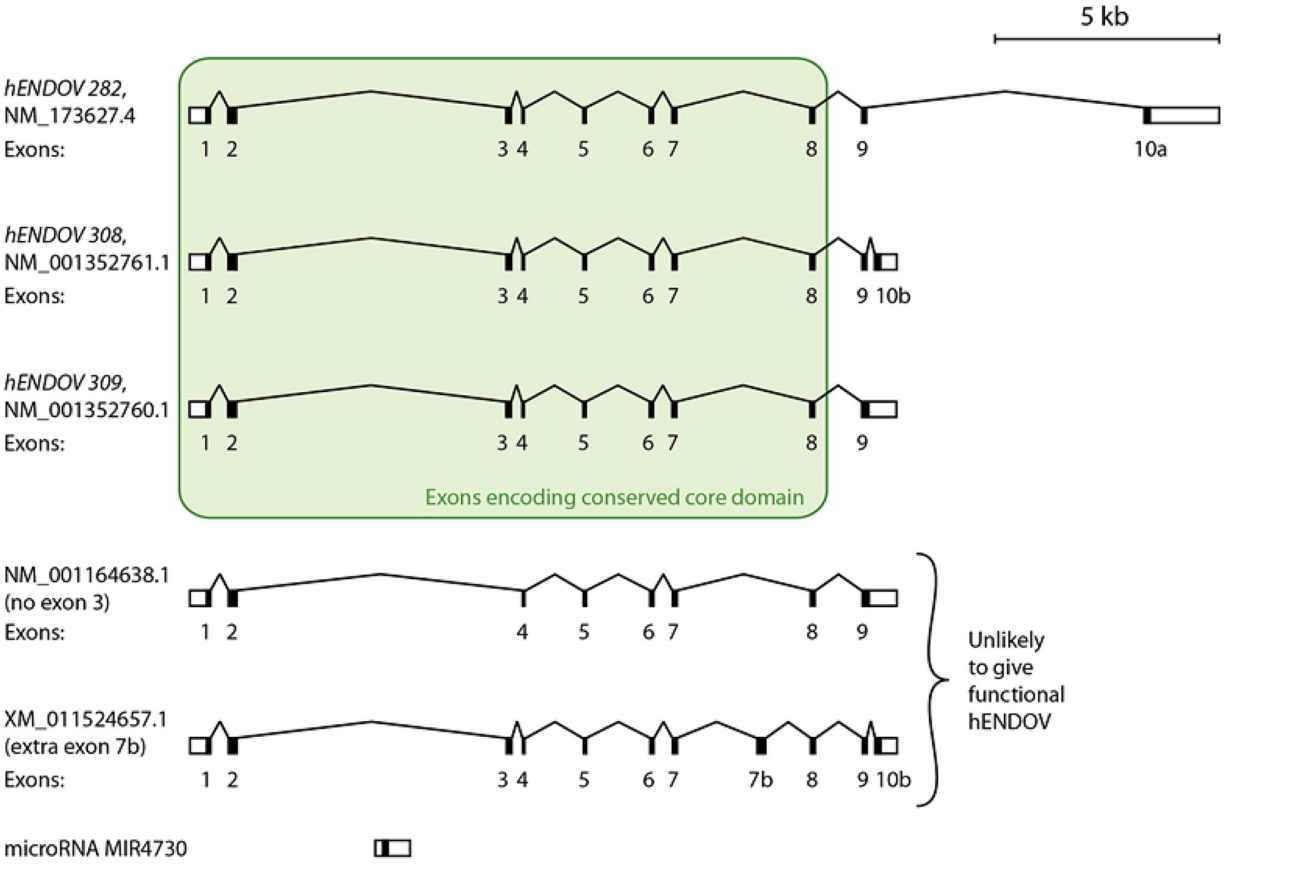
Transcript variants of *hENDOV*. Among more than 40 predicted transcripts for *hENDOV*, only three; *hENDOV 282* (NCBI RefSeq identifier NM_173627.4), *hENDOV 308* (NM_001352761.1) and *hENDOV 309* (NM_001352760.1) comprise all exons encoding the conserved catalytic core (green box, exons 1 to 8) and no more than one or two additional 3’ exons. The two lower transcripts shown in the figure are examples of other transcripts that either lack important exons encoding the catalytic core, or have additional non-conserved exons encoding segments that are likely to disrupt the structure and function of the protein. The *hENDOV* gene is located on chromosome 17q25.3, on the positive strand, with a length of approximately 25,000 base pairs (see scale bar above the gene structure). The lengths of the exons are not shown at the correct scale. 5’-untranslated regions (5’-UTRs) and 3’-UTRs are shown as white boxes in exon 1 and the most 3’ exon, respectively, for each transcript. *MIR4730*, a non-coding RNA in the microRNA class, transcribed in the same direction as *hENDOV*, is located in intron 2 (lowest transcript).

The core domain of ENDOV, including the seven-stranded β-sheet and two C-terminal α-helices (α6 and α7 in hENDOV) [10], is conserved in all three domains of life [2,10] and appears to be absolutely necessary for enzyme function. There are currently listed no less than 43 alternative splice variants for *hENDOV* in the NCBI RefSeq database, and 38 of these are predicted to encode protein, while Ensembl and UniProt list 22 and 5 protein variants, respectively. Eight exons, all highly conserved in mammals and in more distantly related eukaryotes [14], encode the hENDOV core domain (Fig 1), but the majority of the predicted transcripts in public databases lack one or several of these eight core exons and are highly unlikely to be translated into functional hENDOV proteins. For example, many transcripts are lacking exon 3 (Fig 1) which contains a segment encoding a β-strand and an α-helix (β3 and α3 in hENDOV) that is highly conserved in ENDOV from bacteria and archaea to mammals. In addition, many of the predicted transcripts have additional, non-conserved exons spliced in between conserved exons 1 to 8, for example an exon 7b between exons 7 and 8 (Fig 1). A putative hENDOV variant with exon 7b will have an insertion of 61 residues in the middle of highly conserved α7, and is also likely to be misfolded and non-functional. Only three transcripts contain all conserved exons 1 to 8, a common exon 9, and no additional unconserved exons in the core region. They differ in the length of the protein coding part of exon 9 and two have two alternative 3’ exons, exons 10a and 10b (Fig 1 and S1 Fig) (NM_173627.4 = *hENDOV 282*, NM_001352761.1 = *hENDOV 308* and NM_001352760.1 = *hENDOV 309*). Of these, referred to as “full-length” hereafter, the hENDOV 282 isoform is the one that has been used in previous research [11,12,28,29]. It is the default “canonical sequence” in UniProt and the APPRIS [30] principal isoform of the gene. hENDOV 309 is the variant that is conserved in mammals and other animals, such as mouse, pig, chicken and sea urchin [14], but neither hENDOV 308 nor hENDOV 309 are listed as transcripts/proteins in the Ensembl or UniProt databases.

The Genotype-Tissue Expression (GTEx) project portal [31] shows tissue-specific gene expression data for most human genes, for more than 50 tissues and hundreds of individuals. RNA-seq transcript data are also available for *hENDOV*, but the analysis at the GTEx portal is not optimal due to the large amount of non-functional computationally predicted splice variants for *hENDOV*. For example, of the three full-length variants of *hENDOV* discussed above, only *hENDOV 282* is included in the Ensembl list of transcripts that have been used in the GTEx isoform expression analysis. Expression analysis is also complicated by expressed RNA that matches the DNA segment between exons 2 and 3, in particular in brain tissue. These transcripts may be related to expression of the *MIR4730* miRNA (Fig 1) [32]. While there is currently no indication that these transcripts in any way are related to *hENDOV* expression, they are counted as contributing to expression of *hENDOV* in GTEx portal due to the suboptimal quality of the Ensembl transcript data.

A careful investigation of GTEx transcripts that map to exons 1 to 9 as well as 10a and 10b, and transcripts that span the junctions between these exons, suggests that *hENDOV* is expressed at similar and quite low levels in all investigated tissues, except for testis, where expression is roughly three times higher. Transcripts with splicing of exon 2 to 4, that is, skipping of exon 3 (Fig 1), is very common, for example, the ratio of exon 2–3 to exon 2–4 splicing is 1.3 in testis, 0.7 in kidney and 0.6 in skeletal muscle. In the testis data, RNA-seq reads that span exon junctions 5–6 (i.e. reads that support splicing of exon 5 to 6), 6–7, and 8–9 have roughly the same frequency, while 9–10a and 9–10b are slightly lower. All in all, GTEx testis data from 259 individuals suggests that of the transcripts comprising exons 8 and 9, 35% have 3’ splicing as in *hENDOV 309*, 25% as in *hENDOV 282*, and 40% as in *hENDOV 308*. Splicing in other tissues appears to be similar, with all three variants present and none of them dominating by large amounts.

### *hENDOV* transcript levels

**Fig 2:**
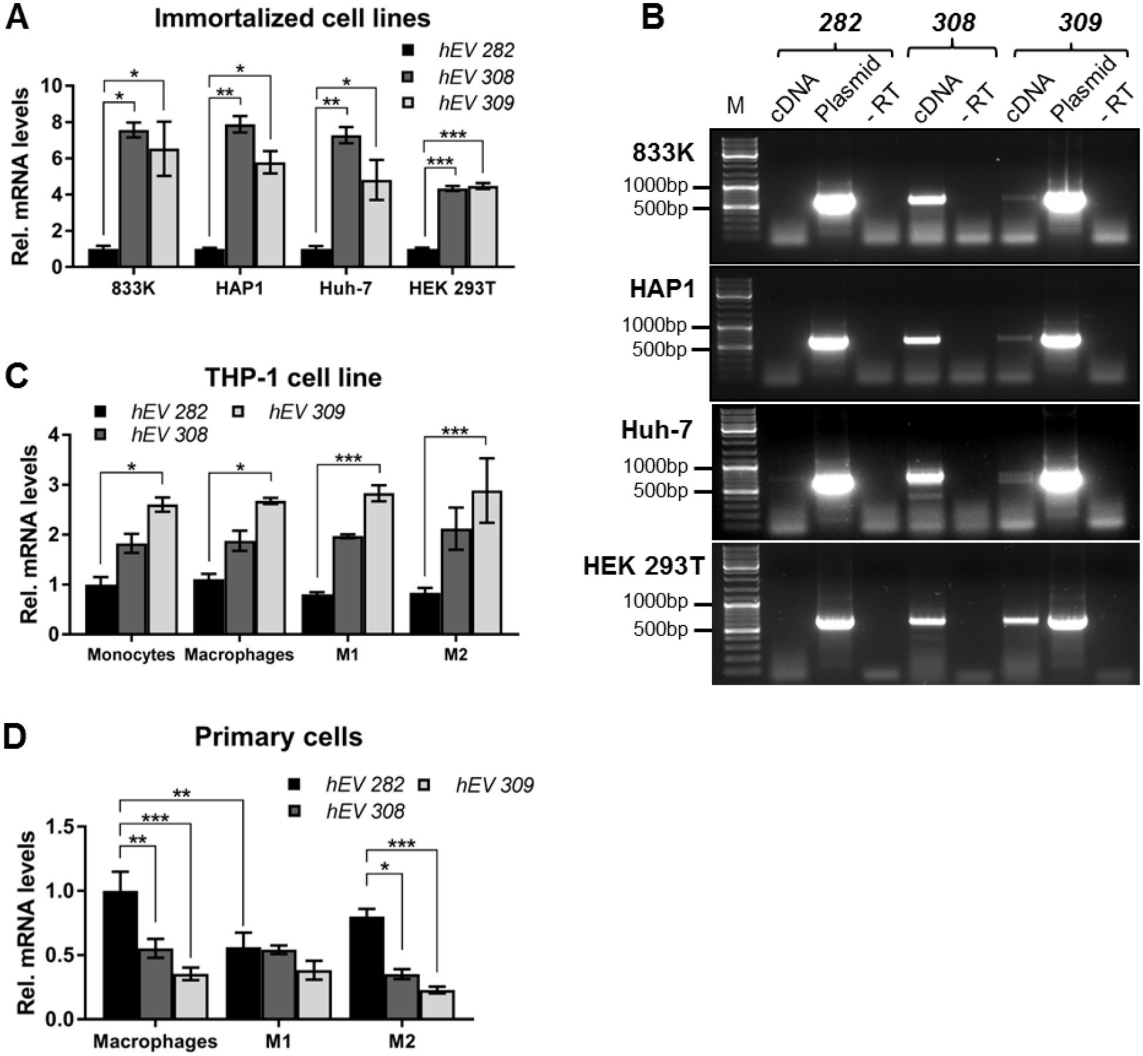
Transcript levels of *hENDOV*. (A) *hENDOV* transcript levels were analyzed by RT-qPCR in 833K, HAP1, Huh-7 and HEK 293T cell lines. Primers specific for *hENDOV 282 (hEV 282)*, *hENDOV 308 (hEV 308)* or *hENDOV 309* (*hEV 309*) 3’-termini were used. The amount of each isoform is relative to *hENDOV 282* in each cell line. One-way ANOVA with a post hoc Tukey’s test was used to calculate the statistical significance in each cell line (n = 3–6). *p < 0.05; **p < 0.01; ***p < 0.001. (B) *hENDOV* transcript presence was analyzed by PCR in 833K, HAP1, Huh-7 and HEK 293T cell lines. An exon 2/3 junction primer was used in pair with 3’-specific primers for *hENDOV 282 (282)*, *hENDOV 308 (308)* or *hENDOV 309* (*309*). Plasmids for hENDOV 282 and hENDOV 309 were included as positive controls for the specific PCRs. A cDNA sample without reverse transcriptase (-RT) added in the reaction was included as negative control. PCR products were analyzed by gel electrophoresis and sizes in base pairs (bp) of the DNA marker (M) are indicated. (C) *hENDOV* transcript levels were analyzed by RT-qPCR in the THP-1 cell line (monocytes, monocytes differentiated to macrophages, and macrophages polarized to M1 or M2) with 3’-specific primers, as described in A. The amount of each isoform is relative to *hENDOV 282* in monocytes. Two-way ANOVA with a post hoc Tukey’s test was used to calculate the statistical significance (n = 3–4); analysis of simple effects (*p < 0.05; ***p < 0.001). (D) *hENDOV* transcript levels were analyzed by RT-qPCR in primary human macrophages and macrophages polarized to M1 or M2 with 3’-specific primers, as described in A. The amount of each isoform is relative to *hENDOV 282* in macrophages. Two-way ANOVA with a post hoc Tukey’s test was used to calculate the statistical significance (n = 7–8); analysis of simple effects (*p < 0.05; **p < 0.01; ***p < 0.001).

To compare the levels of the three full-length *hENDOV* isoforms, RT-qPCR with primers specific for each 3’-end was performed. In four different human cell lines; 833K (from a metastatic testicular germ cell tumor), HAP1 (from chronic myelogenous leukemia), Huh-7 (from a hepatocyte derived cellular carcinoma) and HEK 293T (from human embryonic kidney cells), we found lowest levels of the *hENDOV 282* transcript (Fig 2A). The *hENDOV 308* and *hENDOV 309* isoforms were expressed at a similar level and were significantly higher (4 to 8-fold increase) than *hENDOV 282* in all four cell lines tested (Fig 2A). It appears that the *hENDOV 282* isoform commonly used for analyses and research is the one with lowest mRNA expression in the human cell lines tested.

Having established that cells make *hENDOV* transcripts with different 3’-termini, and having in mind the abundance of incomplete *hENDOV* transcripts found in databases, we evaluated the presence of full-length *hENDOV* transcripts. A standard PCR was performed with a forward primer spanning the exon 2/3 junction together with either of the three 3’-specific reverse primers. PCR products of expected sizes were obtained for *hENDOV 308* (628 bp) and *hENDOV 309* (651 bp) when analyzing cDNAs from 833K, HAP1, Huh-7 and HEK 293T (Fig 2B). For *hENDOV 282* no visible PCR product was obtained using 833K, HAP1 nor HEK 293T cDNAs and only a faint band could be seen for Huh-7 (expected size 630 bp, Fig 2B), which is in agreement with the low expression of this isoform found in RT-qPCR (Fig 2A). DNA sequencing of the PCR products for *hENDOV 308* and *hENDOV 309* verified the presence of all exons. Additional PCR products of shorter sizes was seen for *hENDOV 308* in 833K, Huh-7 and HEK 293T cells (~550 bp) and for *hENDOV 309* in Huh-7 cells (~620 bp) which could originate from alternative incomplete transcripts.

Upon activation, inflammatory cells express nitric oxide (NO) synthases to generate NO to combat the threats [33]. NO has the potential to induce deamination and one may expect a role for hENDOV under such conditions. We therefore extended the RT-qPCR analyses to include the monocytic cell line THP-1. Also in these cells, the level of *hENDOV 282* mRNA was significantly lower than *hENDOV 308* and *hENDOV 309* mRNAs (2 to 3-fold). We checked if the transcript levels of the different isoforms changed upon differentiation of THP-1 monocytes into macrophages and polarization to M1 (pro-inflammatory) and M2 (anti-inflammatory) macrophages. RT-qPCR analyses showed that macrophages, M1 and M2 cells had the same low amount of *hENDOV 282* transcript and higher of *hENDOV 308* and *hENDOV 309* (2 to 3-fold increase; Fig 2C). There was a significant main effect of isoforms (F (2, 30) = 40.24, p < 0.0001, ***) without interaction. Simple main effects analyses showed *hENDOV 282* transcript levels were significantly lower compared to *hENDOV 309* in monocytes (p = 0.0338, *), macrophages (p = 0.0408, *); M1 macrophages (p = 0.0004, ***) and M2 macrophages (p = 0.0003, ***) (Fig 2C).

Next we did similar experiments with primary macrophages isolated from healthy human blood donors. In these cells, the *hENDOV 282* mRNA level was significantly higher than *hENDOV 308* and *hENDOV 309* levels (2-fold increase; Fig 2D), contrary to what was seen in the monocytic cell line THP-1 (Fig 2C). Upon polarization to M1 macrophages, *hENDOV 282* level was reduced by half relative to unstimulated macrophages, whereas *hENDOV 308* and *hENDOV 309* levels did not change (Fig 2D). In M2 polarized cells, the *hENDOV 282* isoform level did not change relative to macrophages (Fig 2D). There was a significant interaction of isoforms and polarization (F (4, 60) = 3.282, p = 0.0169, *). Simple main effects analyses showed that macrophages *hENDOV 282* transcript level was significantly higher compared to macrophages *hENDOV 308* (p = 0.0012, **), *309* (p = 0.0001, ***) and M1 *hENDOV 282* levels (p = 0.0017, **). M2 *hENDOV 282* transcript level was significantly higher compared to M2 *hENDOV 308* (p = 0.0109, *) and *309* levels (p = 0.0003, ***) (Fig 2D). It thus appears that hENDOV isoforms are differently regulated by inflammatory state in monocytic primary cells.

### Endogenous hENDOV protein isoforms

**Fig 3:**
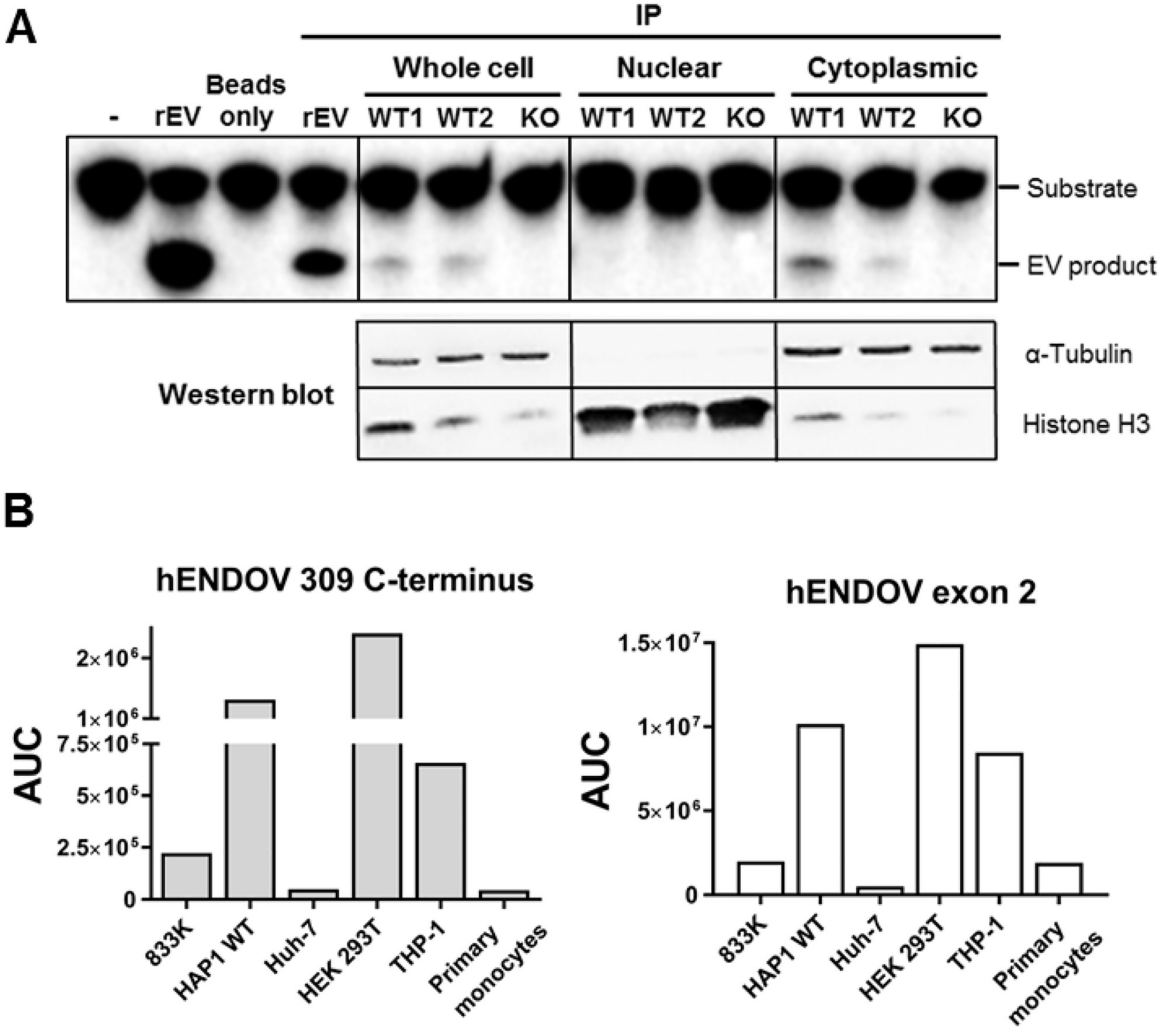
Endogenous hENDOV protein. (A) Whole cell protein lysates, nuclear and cytoplasmic fractions from HAP1 cells (two different wild type clones = WT1, WT2 and *hENDOV*^-^ = KO) were subjected to immunoprecipitation (IP) with a hENDOV antibody. The immunoprecipitated materials were tested in hENDOV activity assays using an inosine-containing single-stranded RNA substrate (ssIIUI) (upper panel). Cleaved fragments (EV product) were separated from intact substrates by polyacrylamide gel electrophoresis. The sample without hENDOV (-) represents intact substrate. Recombinant hENDOV (rEV) was used as a positive control in the IP and activity assays. To verify the quality of the subcellular fractionation, whole cell protein lysates, nuclear and cytoplasmic fractions were subjected to western blot analysis (bottom panels). Antibodies against α-Tubulin and Histone H3 were used as cytoplasmic and nuclear markers, respectively.(B) Detection of hENDOV peptides by mass spectrometry (MS). Protein lysates from 833K, HAP1 WT, Huh-7, HEK 293T, THP-1 cells as well as primary monocytes were subjected to immunoprecipitation with a hENDOV antibody and analyzed by MS. Amounts of peptides detected are shown as area under curve (AUC). Left panel shows detected peptides of hENDOV 309 (GDSGESSGEGQPPQDHSPGPR [273, 293 amino acids (aa)]) and right panel shows exon 2 peptides (DPAFSGLQR [39, 47 aa]).

As different *hENDOV* transcripts exist in cells, we next analyzed which protein isoforms are found. No specific signal for hENDOV could be detected in ordinary western blot analyses of whole cell protein lysates from various cell lines. Previously, extensive chromatographic purification was needed to obtain hENDOV signals in western blot analyses, probably due to low levels of endogenous hENDOV [13]. Nevertheless, all three recombinant hENDOV proteins (hENDOV 282, 308, 309) were detected by western blot after immunoprecipitation, demonstrating that the antibody can recognize all three isoforms (S2A Fig). Next, immunoprecipitation of endogenous hENDOV in whole cell protein lysates from various cell lines was performed. Although this approach also failed to give specific signals for hENDOV in western blot analyses, we were able to demonstrate inosine-specific ribonuclease activity in HAP1 wild type lysates with endogenous hENDOV immunoprecipitated. The size of the cleavage product was the same as for recombinant hENDOV and moreover, this activity was absent in imunoprecipitated HAP1 *hENDOV*^-^ lysates, strongly suggesting that hENDOV is responsible for this cleavage (Fig 3A, upper panel). Further characterization included subcellular protein fractionation in HAP1 cells prior to immunoprecipitation. Interestingly, the inosine-specific ribonuclease activity was seen in the cytoplasmic fractions, and not in the nuclear fractions (Fig 3A, upper panel). Presence of α-Tubulin (cytoplasmic) and Histone H3 (nuclear) confirmed adequate fractionation (Fig 3A, lower panels).

A more sensitive method to precisely identify hENDOV isoforms is targeted quantitative proteomics by mass spectrometry, employing parallel reaction monitoring (PRM). Due to its high selectivity and sensitivity, PRM enables the detection of specific peptides from preselected protein targets in complex samples. We analyzed whole cell protein lysates from HAP1 and THP-1 cells with emphasis on the three different C-termini of hENDOV. No signals above the background level were obtained, in agreement with the low level of endogenous hENDOV already described. To increase the amount of input material, we immunoprecipitated hENDOV from 833K, HAP1, Huh-7, HEK 293T, THP-1 and primary macrophages lysates prior to PRM analyses. This increased the amount of specific signals and a C-terminal peptide corresponding to hENDOV 309 (GDSGESSGEGQPPQDHSPGPR [273, 293 amino acids (aa)]) was found in all 5 cell lines as well as in the primary monocytes, (Fig 3B, left panel). Interestingly, neither hENDOV 282 (GDSGESSALC [273, 282 aa]) nor hENDOV 308 (GDSGESSGGAPSPQR [273, 287 aa]) C-termini specific peptides were detected above background levels by this method. A peptide originating from exon 2 (DPAFSGLQR [39, 47 aa]) was found in all samples except in HAP1 *hENDOV*^-^ cells (Fig 3B, right panel). For both peptides (309 and exon 2) highest signals were found in HEK293T, HAP1 and THP-1 cells (Fig 3B), probably reflecting more hENDOV protein in these cells. However, due to the experimental setup including immunoprecipitation, direct comparison of quantity is imprecise, even with the use of equal amount of input material in all samples. It thus appears that cells produce solely the hENDOV 309 protein, despite that these transcripts exist for hENDOV 308 and hENDOV 282.

### Analyses of recombinant hENDOV protein isoforms

**Fig 4:**
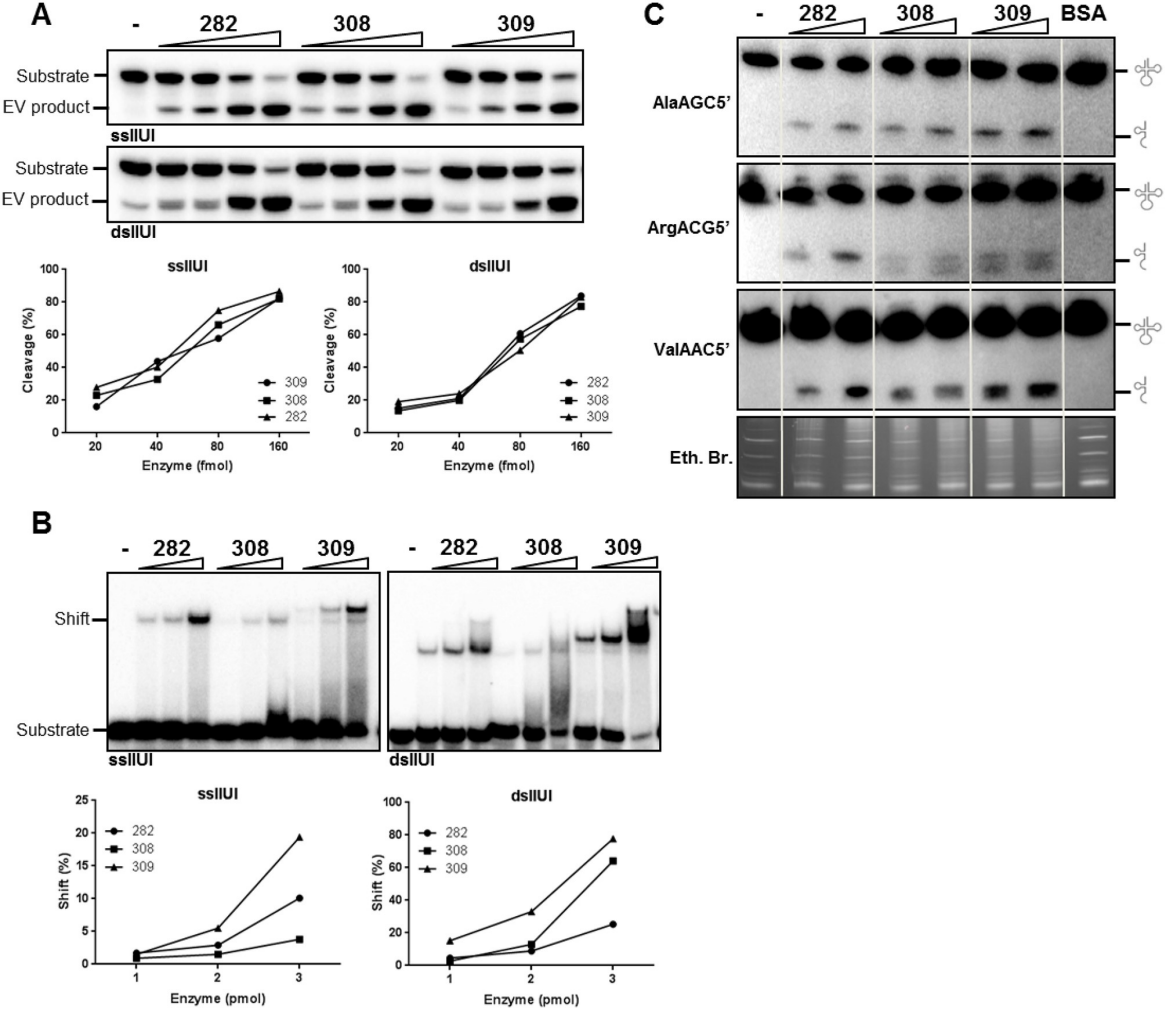
Enzymatic properties of recombinant hENDOV proteins. (A) RNA cleavage activity of hENDOV 282 (282), hENDOV 308 (308) and hENDOV 309 (309) enzymes. Increasing amounts of enzyme (20, 40, 80 and 160 fmol) were incubated with a single stranded (ss; upper) or double-stranded (ds; lower) RNA substrates containing inosine and reaction products were analyzed by denaturing gel electrophoresis. Quantification of the cleavage activity by ImageQuant TL software (n = 3) is shown below. (B) Electrophoretic mobility shift assay using hENDOV 282 (282), hENDOV 308 (308) and hENDOV 309 (309) enzymes (1, 2 or 3 pmol) and ss (left panel) or ds (right panel) RNA substrates containing inosine. Reactions were incubated on ice prior to separation of enzyme-substrate complexes from unbound substrate on native polyacrylamide gels. Quantification of band shifts by ImageQuant TL software (n = 3) is shown below. (C) Representative images of northern blot analyses of total RNA from HAP1 cells (WT) incubated with recombinant hENDOV 282 (282), hENDOV 308 (308) and hENDOV 309 (309) enzymes (75 and 150 pmol). The probes used recognize AlaAGC5’, ArgACG5’ or ValAAC5’ tRNAs. Sample without enzyme (-) or with 150 pmol BSA were included as controls. Equal loading is shown by ethidium bromide (Eth. Br.) staining of the gel (lower panel). Glyphs to the right of the membranes indicate full-length and fragmented tRNA species.

Although MS did not reveal C-terminal peptides for all the three hENDOV isoforms, we cannot fully exclude their presence in certain cells or under certain conditions. We therefore aimed to determine biochemical properties of the three hENDOV protein isoforms. The proteins were purified after expression in *E*. *coli* (S2B Fig) and analyzed with respect to enzymatic activity. When assayed with ss- or ds-RNA substrates containing inosine, the enzymes were equally active (Fig 4A), suggesting that the C-terminus of hENDOV is not critical for catalysis.

We next analyzed substrate binding of hENDOV by electrophoretic mobility shift assay (EMSA). All three isoforms showed shifts when using the inosine-containing ss- and ds-RNA substrates. hENDOV 309 showed stronger shifts than hENDOV 282 and hENDOV 308 with both substrates (Fig 4B). For the ssRNA substrate, hENDOV 308 had weaker affinity than hENDOV 282. Under the EMSA conditions used, no cleavage of the inosine-substrates was seen, demonstrating the experiments show substrate and not product binding (S2C Fig). Interestingly, a truncated version lacking the unstructured C-terminus but containing the whole hENDOV-core segment (amino acids 1–250 [14]; S1 and S2B Figs) completely lost the RNA binding affinity and also inosine-ribonuclease activity was strongly reduced (S2D and S2E Fig). These results suggest that the C-terminus of hENDOV makes contacts with the RNA substrate and influences enzyme binding and activity.

Previous research has shown that recombinant hENDOV 282 cleaves inosine-containing tRNAs *in vitro* [12]. We tested tRNA cleavage by the three isoforms by northern blot analyses. We found tRNA fragments at comparable levels for all three hENDOV enzymes using probes for AlaAGC5’, ArgACG5’ and ValAAC5’ (Fig 4C). For Arg tRNA a double band is seen for hENDOV 308 and hENDOV 309, but not hENDOV 282 (Fig 4C). As no evidence exists for hENDOV cleaving RNA independently of inosine [12], the lower band is probably a result of contaminating nucleases following as high amounts of enzymes are used in these assays.

### Subcellular distribution of the GFP-hENDOV fusion proteins

**Fig 5:**
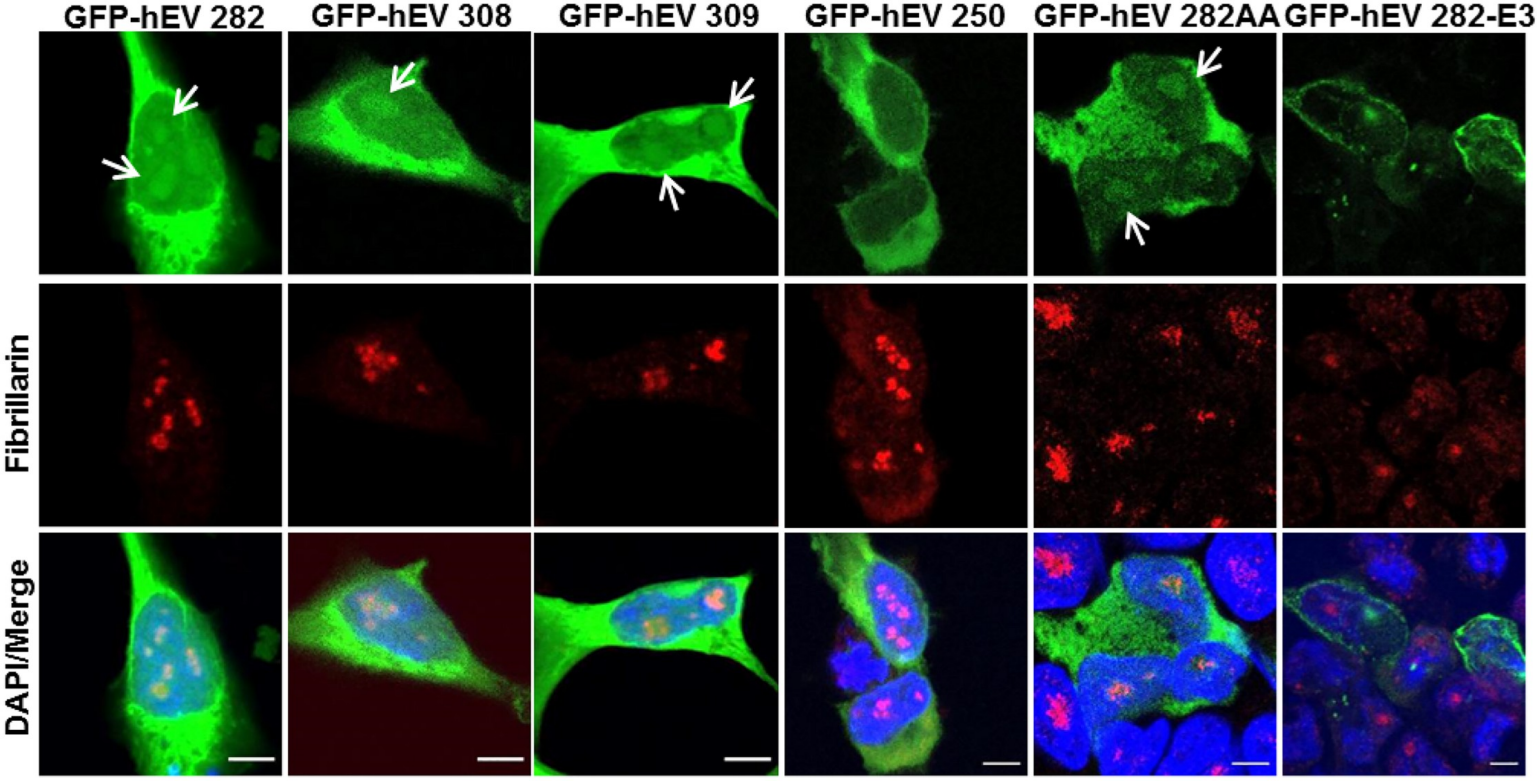
Subcellular distribution of ectopically expressed hENDOV proteins. HEK 293T cells were transiently transfected for expression of GFP fused hENDOV isoforms (GFP-hEV 282, GFP-hEV 308, GFP-hEV 309, GFP-hEV 250, GFP-hEV 282AA and GFP-hEV 282-E3) (green). Cells were stained with a Fibrillarin antibody to visualize nucleoli (red). Nuclei are shown by DAPI staining (blue) and colocalization is shown in merged images. Arrows show dots representing nucleolar localization of GFP-hEV isoforms. Confocal images were acquired using an x40 oil objective, with 6–8x zoom (Leica SP8). Bar 5μm.

Ectopically expressed hENDOV 282 is found in the cytoplasm of human cells with some nucleolar staining [14]. Targeting of proteins to nucleoli depends on positively charged amino acids (clustered arginines and lysines) with a high isoelectric point (pI) [34] which are also features of the more defined nuclear localization signal (6–10 basic amino acid residues) [35]. Analysis of the hENDOV amino acid sequence by WoLF PSORT protein localization prediction does not show any particular localization motifs. Manual inspection of the C-termini part that differs between the three hENDOV isoforms, identifies no argines/lysines in hENDOV 282, three in hENDOV 308 and five in hENDOV 309 (S1 Fig). To test if this influences the subcellular distribution, human HEK 293T cells were transiently transfected for expression of the hENDOV isoforms fused to GFP. We found strong cytoplasmic staining for all three fusion proteins with minimal signals in the nucleus (Fig 5). A closer inspection revealed GFP-hENDOV 282 signals in the nucleus overlapping with Fibrillarin, indicating nucleolar localization (Fig 5), as previously reported [14]. This was also the case for GFP-hENDOV 308 and GFP-hENDOV 309 isoforms (Fig 5). We tested the C-terminal truncated version of hENDOV (GFP-hENDOV 250) which was found mainly in cytoplasm with no visible nucleolar accumulation (Fig 5). Western blot analyses showed that all GFP-hENDOV proteins were expressed as fusion proteins both in cytoplasm and nuclei (S2G Fig). However, in western blot of nuclear fractions, GFP-hENDOV 250 level was low, in agreement with no/weak microscopic nuclear signal for this variant. As mentioned before, amino acids 1–250 correspond to full-length *E*. *coli* EndoV, but it seems that in human cells the extended C-terminus is important for normal function and sorting of hENDOV.

Another structural difference between the bacterial and human enzyme is the presence of an extra α-helix (α4) in hENDOV. To see if subcellular sorting relies on this helix, we analyzed a mutant with a disrupted helix (E171A, R174A; referred to as hENDOV 282AA). The hENDOV 282AA mutant showed similar cytoplasmic and nucleolar staining as the wild type enzymes, suggesting that the α4 helix is not important for intracellular localization. The recombinant version of hENDOV 282AA had same enzymatic activity as wild type hENDOV 282 (S2F Fig).

Many *hENDOV* transcripts found in the databases lack exon 3 which we believe will encode nonfunctional proteins due to the lack of crucial amino acid residues. We analyzed the sorting of a GFP-hENDOV 282 exon 3-deletion mutant (referred to as hENDOV 282-E3) and found barely visible signals in the cytoplasm with no nucleolar staining (Fig 5 and S2G Fig). Low expression and altered sorting suggests that hENDOV without exon 3 is likely dysfunctional.

**Fig 6:**
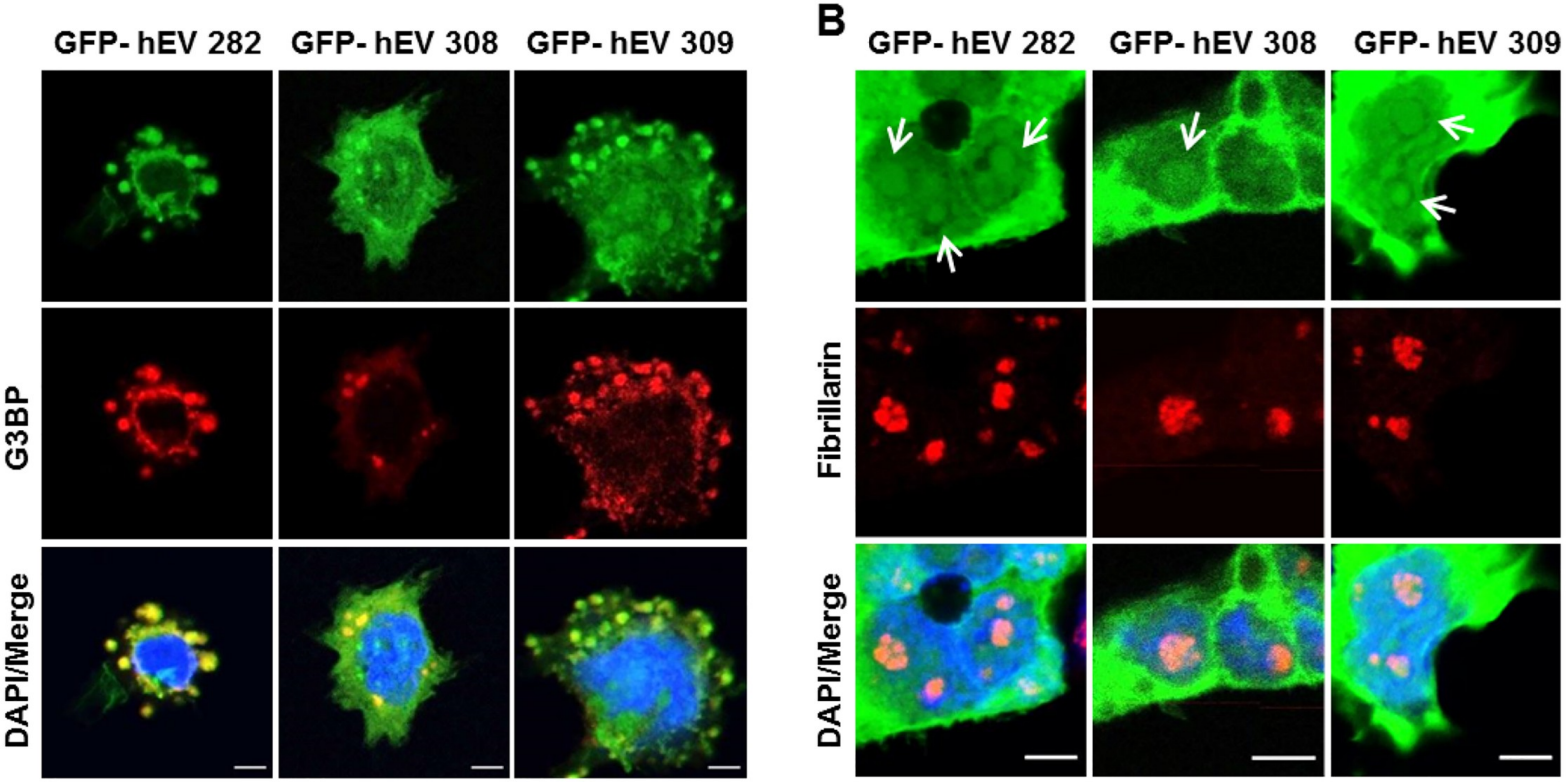
Subcellular localization of hENDOV proteins during oxidative stress. Localization of GFP fused hENDOV isoforms (GFP-hEV 282, GFP-hEV 308, GFP-hEV hENDOV 309) (green) in stress granules (A) and nucleoli (B) after arsenite exposure (0.5 mM, 30 min). HEK 293T cells were transiently transfected for expression of GFP fused hENDOV. Cells were stained with G3BP1 (A; red) or Fibrillarin (B; red) antibodies to visualize stress granules and nucleoli, respectively. Arrows show dots representing nucleolar localization of GFP-hEV isoforms. Nuclei are shown by DAPI staining (blue) and colocalization is shown in merged images. Confocal images are acquired using an x40 oil objective, with 8–10x zoom (Leica SP8). Bar 5μm.

Upon various stress, hENDOV 282 relocalizes to cytoplasmic stress granules [13]. After arsenite treatment, also the hENDOV 308 and hENDOV 309 isoforms were found in stress granules (Fig 6A). Nucleoli organization may also be affected by certain stress, often leading to disruption and/or morphological and proteomic changes [36]. We analyzed the GFP-hENDOV isoforms distribution in nucleoli after arsenite treatment and found all three isoforms (282, 308 and 309) signals could still be found overlapping with fibrillarin (Fig 6B). Thus, the most distal part of the hENDOV C-termini seems disposable and proper subcellular sorting seems to rely on information from other motifs/domains embedded in the common part of the hENDOV C-terminus. In sum, all three hENDOV isoforms have a similar tripartite cellular distribution; cytoplasm, nucleoli and stress granules upon stress.

## Discussion

Endonuclease V homologs constitute a family of evolutionary conserved nucleases with specificity for inosines in nucleic acids. The *in vitro* activity is well established, yet the *in vivo* function remains unknown. To learn more about hENDOV, we performed transcript and protein analyses as well as an update on available information in databases. As previously reported, public databases show a multitude of different *hENDOV* transcripts, and most of these transcripts are likely incomplete [14]. However, three different isoforms are present containing all information needed to make the conserved hENDOV core together with an extended C-terminus. In fact, these proteins are similar for amino acids 1–279 and differ only in the 3–30 last residues. By 3’-specific RT-qPCR and PCR we show that all three variants are transcribed in various cells, however, at the protein level we find evidence only for the hENDOV 309 isoform. Identical splicing as the one occurring in the 309 isoform is found in other vertebrates such as mouse and chicken [14].

Until now, all research done with ectopically expressed hENDOV used the hENDOV 282 isoform [11–14,37]. Interestingly, we found significantly lower mRNA level for this isoform than for the other two isoforms *hENDOV 308* and *hENDOV 309*. This was consistent in all 5 cell lines tested. In agreement, MS analyses did not detect any hENDOV 282-specific peptides and the only confirmed protein isoform was hENDOV 309. The 309 protein has a molecular weight of 33.7 kDa compared to 30.8 kDa for 282 which could explain the slower migrating band in western after purification of endogenous hENDOV [13]. Surprisingly, no hENDOV 308-specific peptides were found after MS, despite that transcripts levels of *hENDOV 308* and *hENDOV 309* were always equally higher than those of hENDOV 282. How the translational machinery can select between these very similar transcripts differing in coding information only for less than 30 C-terminal amino acid residues is unknown. Of note, MS-signals for hENDOV 282 and hENDOV 308 peptides were detected in all the cell lines analyzed, including HAP1 lacking ENDOV. However, careful examination of the MS spectra, showed that these signals corresponded to unspecific peptides with the same retention properties as the hENDOV peptides, and thus interfered with the analyses. Unfortunately, due to limitations in selection of unique peptides (due to the short C-terminus of each isoform), we had no opportunity to improve the specificity of the experiment. Thus, we cannot exclude that a certain amount of hENDOV 282/308 proteins are present in cells, but we are not able to distinguish those from the background.

After having analyzed immortalized cells for *hENDOV* isoforms, we did similar RT-qPCR experiments in human primary cells, namely macrophages. In unstimulated macrophages the levels of the *hENDOV* transcripts were reversed relative to the immortalized cell lines, with *hENDOV 282* mRNA at higher levels than *hENDOV 308* and *hENDOV 309* mRNA. The same was found in M2 macrophages, whereas in M1 macrophages the three isoforms were found at comparable levels. Interestingly, upon polarization to M1 macrophages, *hENDOV 282* mRNA was reduced to half the level of that in macrophages, whereas such a decrease was not seen upon polarization to M2 macrophages. Whether this reflects reduced expression or increased decay of *hENDOV 282* mRNA in M1 macrophages remains to be determined. In M1 cells NO synthetases are activated to produce NO in the defense against invading bacteria and virus [38]. NO is a reactive metabolite that may enhance deamination thus leading to increased inosine levels. Also ADAR p150 deaminase is induced upon infection/inflammation, further increasing inosine levels [39]. Thus, a role for hENDOV under such conditions is likely and one may argue for the need of both to increase hENDOV activity to remove damaged transcripts and to reduce hENDOV activity to avoid unwanted degradation of ADAR-induced inosine-containing transcripts. Adjusting *hENDOV* transcript levels could be a way for the cell to handle the specific conditions. Altogether, our data show that the levels and different types of *hENDOV* transcripts in cells are subjected to regulation which may reflect that cells need various types/amounts of hENDOV depending on growth/environmental conditions.

We show that different *hENDOV* transcripts exist in cells, and consequently, one may expect protein isoforms with distinct functions. We examined biochemical properties of the corresponding recombinant proteins and found that inosine-cleaving activity was comparable for the three isoforms. However, binding affinities varied as hENDOV 309 bound stronger and hENDOV 308 weaker than hENDOV 282 to the ss RNA substrate. hENDOV 309 affinity to the ds-RNA substrate was also stronger than for the other two isoforms. Further, the hENDOV 309 shift migrated slower than the hENDOV 308 shift (more than expected for a difference in molecular weight of less than one kDa). This could reflect how the C-terminus of hENDOV 309 interacts with the RNA and contributes to a more stable and slower migrating RNA-enzyme complex. The amino acid composition of hENDOV 308 and hENDOV 309 C-termini are clearly different, and the last thirty amino acids specific for hENDOV 309 have a pI of 5.5 compared to 12.2 for the twenty-nine C-terminal amino acids of hENDOV 308. For hENDOV 282 only three amino acids are specific and therefore more difficult to interpret. On the other hand, the C-termini, for all three variants, are strongly predicted to be structurally disordered which is also the case for ENDOV in other mammals [14]. Further, it was shown that the C-terminus was not at all conserved and is evolving neutrally in rodents [14]. That is, it appears that there is no evolutionary pressure to keep the sequence conserved. Nevertheless, the three hENDOV isoforms have different biochemical properties which could be important for the cell to fine tune hENDOV function.

The three hENDOV isoforms were studied with respect to subcellular distribution. Previous research has shown that hENDOV 282 is located in cytoplasm, nucleoli and stress granules [13]. Also hENDOV 308 and hENDOV 309 fused to GFP had a similar localization, indicating that the 29–30 C-terminal amino acids are not critical for cellular targeting of hENDOV. On the other hand, the C-terminal truncated version hENDOV 250 was not detected in nucleoli. Thus, the whole unstructured C-terminus (from amino acids 250) could be important for hENDOV distribution in cells. It should however be mentioned, that the GFP-signal intensities of the transfected cells were lower for hENDOV 250, hENDOV 308 and hENDOV AA isoforms, and thus multiple frames were accumulated during image acquisition to achieve similar intensity levels as the hENDOV 288 and hENDOV 309 isoforms. Nevertheless, whereas hENDOV 309 and hENDOV AA isoforms were detected in nucleoli after multiple frame accumulations, hENDOV 250 remained undetected.

The whole C-terminus was not only important for subcellular sorting, but turned crucial also for enzymatic properties, as recombinant hENDOV 250 lost RNA binding and inosine-cleavage activity. This emphasizes the significance of this region for the human enzyme contrasting to the situation in *E*. *coli*, where ENDOV lacks this C-terminus but has full activity. Another structural difference between the bacterial and human enzyme is the additional α-helix (α4) [10]. This helix is opposed to the active site and is likely not involved in catalysis as demonstrated with recombinant hENDOV 282AA mutant which was fully active. Moreover, disruption of the helix did not influence cellular sorting. The function of this helix is still unknown but participation in protein-protein interactions is likely.

Alternative mRNA splicing is an essential regulatory mechanism in eukaryotic gene expression that controls proper function of proteins [18]. Considering that mis-splicing can change important physiological protein properties and result in various human diseases, a fine-tuned balance of alternative splicing is essential for human health. It is therefore important to understand regulation, function and levels of different isoforms for a single gene. In this study we have characterized new hENDOV isoforms and suggest that future research in this field should include the hENDOV 309 isoform. Hampered by low endogenous expression levels of hENDOV, further analyses are needed to fully address hENDOV function.

## Supporting information

S1 Fig Amino acid sequences of the three hENDOV isoforms analyzed in the study. Alternating black and blue color highlight the segments encoded by the different exons, red shows splice sites encoded residues and green the two mutated residues in the 282AA mutant: glutamate E171 and arginine R174 to alanines (AA). The last amino acid in the truncated form (Ser250) is underlined. Bold underlining shows the peptides identified in MS.
(TIF)

S2 Fig Analyses of recombinant and GFP fused hENDOV isoforms. (A) Western blot analysis of recombinant hENDOV 282 (282), hENDOV 308 (308) and hENDOV 309 (309) enzymes after immunoprecipitation. Calculated molecular weights: hENDOV 282 (30.8 kDa), hENDOV 308 (33.5 kDa), hENDOV 309 (33.7 kDa). The membrane was probed with a hENDOV antibody (polyclonal; in house). (B) SDS-PAGE of purified hENDOV proteins (2 μg); hENDOV 282 (282), hENDOV 308 (308) and hENDOV 309 (309), (molecular weight as in A), and MBP-hENDOV 282AA (AA; 72.8 kDa) and MBP-hENDOV250 (250; 69.6 kDa). Molecular weight marker (M; in kDa) is shown in the first left lane. (C) Denaturing PAGE analyses of the ss inosine-RNA substrate after EMSA incubation using increasing amount of the hENDOV isoforms (1, 2 or 3 pmol; related to Fig 4B). (D) hENDOV 250 truncated protein (250; 3 pmol) is compromised with respect to inosine-RNA binding as shown by electrophoretic mobility shift assay. Substrates used were ssIIUI (left panel), dsIIUI (middle panel) and ssRNA without inosine (ssCtr; right panel). (E) Inosine-RNA cleavage by hENDOV 250 (250) as shown by activity assay. Increasing amounts of enzyme (40, 200 and 800 fmol) were incubated with the ss IIUI substrate and reaction products analyzed by denaturing gel electrophoresis. hENDOV 282 (hEV) was used as positive control for activity. (F) Inosine-cleavage by hENDOV 282AA (282AA) as shown by activity assay. Increasing amounts of enzyme (20, 40, 80 and 160 fmol) were incubated with ss (upper) or ds (lower) IIUI substrates containing inosine and reaction products were analyzed by denaturing gel electrophoresis. hENDOV 309 (hEV) was used as positive control for activity. (G) Western blot analyses of cytoplasmic (left panels) and nuclear fractions (right panels) of HEK 293T cells transiently transfected to express GFP fused hENDOV isoforms (GFP-hEV). Cells transfected without DNA (Mock) or with a GFP expressing construct (GFP) were included as negative and positive controls, respectively. Molecular weight marker (M; in kDa) is shown in the first left lane. The membranes were probed with GFP (, 1^st^ panel) and hENDOV antibodies (2^nd^ panel) to verify the presence of GFP-hEV isoforms. Antibodies against α-Tubulin (3^rd^ panel) and Histone H3 (bottom, 4^th^ panel) were used to show the quality of the cytoplasmic and nuclear fractions, respectively.
(TIF)

S3 Fig Uncropped images of all gels and blots in the manuscript. (PDF)
