# Supplementary figures and images for: Complex alternative splicing of human Endonuclease V mRNA, but evidence for only a single protein isoform
Source: PLoS One. 2019 Nov 8;14(11):e0225081. doi: 10.1371/journal.pone.0225081 (PMC6839837; doi:10.1371/journal.pone.0225081)

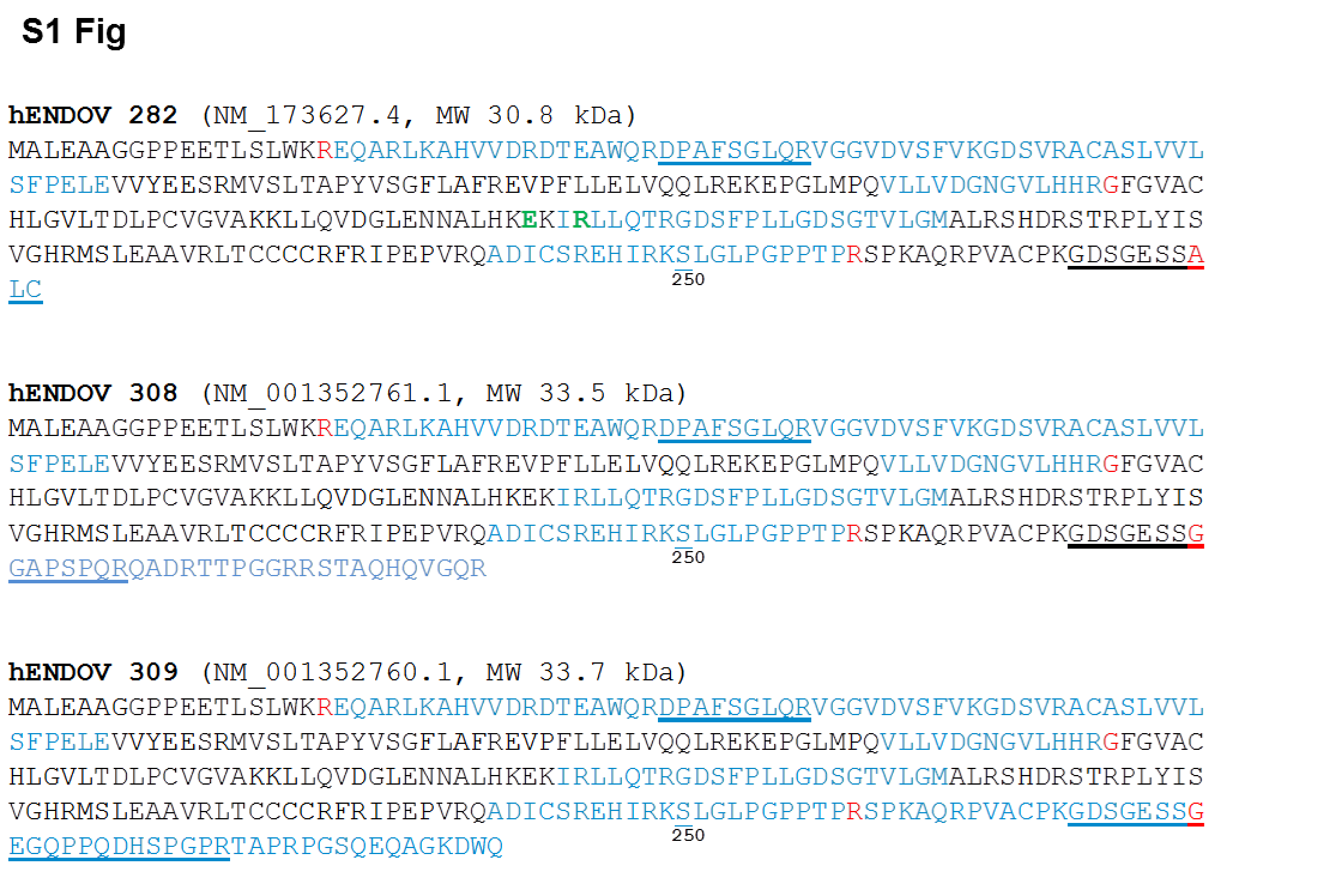

Supplement: S1 Fig — Alternating black and blue color highlight the segments encoded by the different exons, red shows splice sites encoded residues and green the two mutated residues in the 282AA mutant: glutamate E171 and arginine R174 to alanines (AA). The last amino acid in the truncated form (Ser250) is underlined. Bold underlining shows the peptides identified in MS. (TIF) [file pone.0225081.s001.tif]

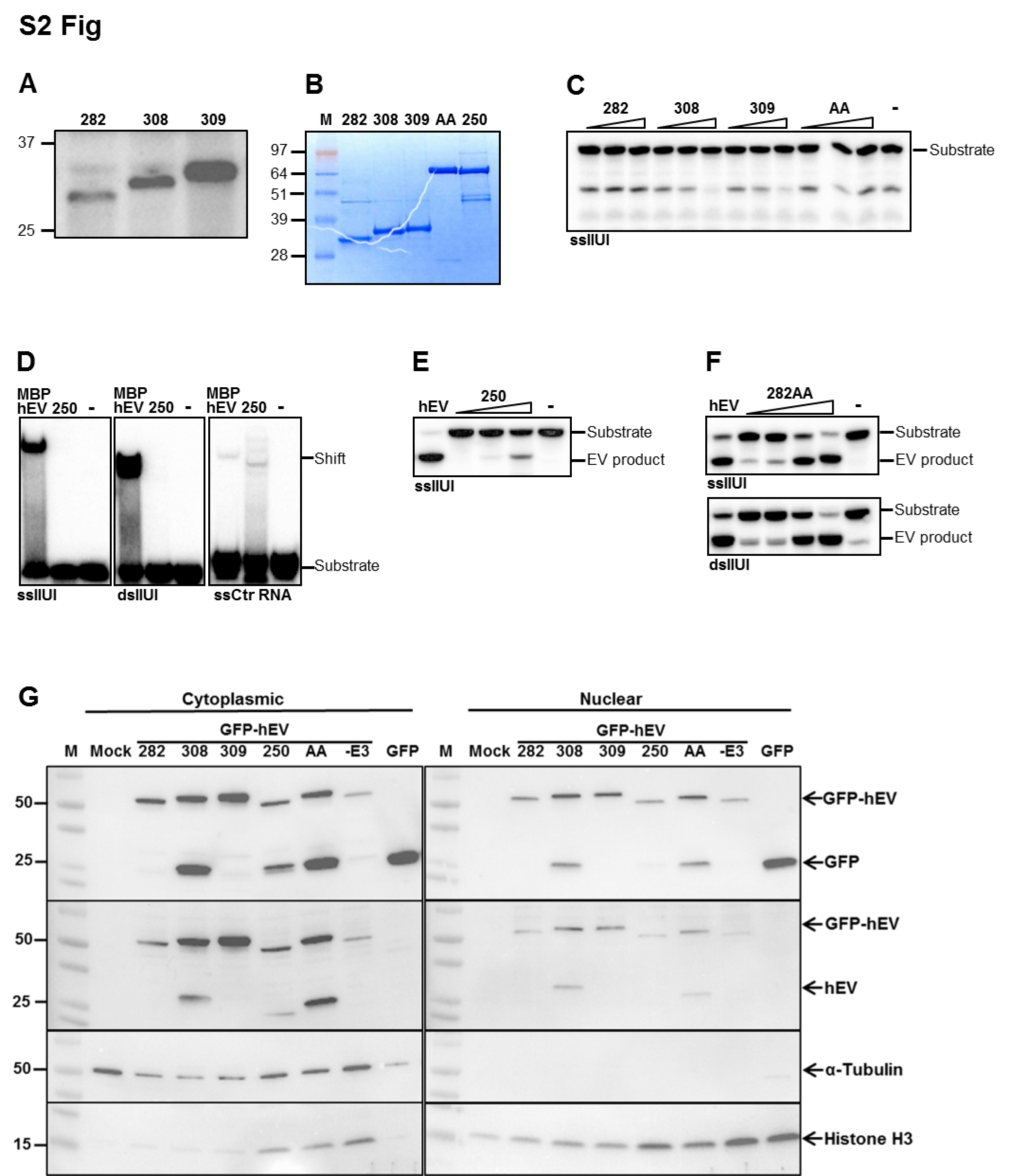

Supplement: S2 Fig — (A) Western blot analysis of recombinant hENDOV 282 (282), hENDOV 308 (308) and hENDOV 309 (309) enzymes after immunoprecipitation. Calculated molecular weights: hENDOV 282 (30.8 kDa), hENDOV 308 (33.5 kDa), hENDOV 309 (33.7 kDa). The membrane was probed with a hENDOV antibody (polyclonal; in house). (B) SDS-PAGE of purified hENDOV proteins (2 μg); hENDOV 282 (282), hENDOV 308 (308) and hENDOV 309 (309), (molecular weight as in A), and MBP-hENDOV 282AA (AA; 72.8 kDa) and MBP-hENDOV250 (250; 69.6 kDa). Molecular weight marker (M; in kDa) is shown in the first left lane. (C) Denaturing PAGE analyses of the ss inosine-RNA substrate after EMSA incubation using increasing amount of the hENDOV isoforms (1, 2 or 3 pmol; related to Fig 4B). (D) hENDOV 250 truncated protein (250; 3 pmol) is compromised with respect to inosine-RNA binding as shown by electrophoretic mobility shift assay. Substrates used were ssIIUI (left panel), dsIIUI (middle panel) and ssRNA without inosine (ssCtr; right panel). (E) Inosine-RNA cleavage by hENDOV 250 (250) as shown by activity assay. Increasing amounts of enzyme (40, 200 and 800 fmol) were incubated with the ss IIUI substrate and reaction products analyzed by denaturing gel electrophoresis. hENDOV 282 (hEV) was used as positive control for activity. (F) Inosine-cleavage by hENDOV 282AA (282AA) as shown by activity assay. Increasing amounts of enzyme (20, 40, 80 and 160 fmol) were incubated with ss (upper) or ds (lower) IIUI substrates containing inosine and reaction products were analyzed by denaturing gel electrophoresis. hENDOV 309 (hEV) was used as positive control for activity. (G) Western blot analyses of cytoplasmic (left panels) and nuclear fractions (right panels) of HEK 293T cells transiently transfected to express GFP fused hENDOV isoforms (GFP-hEV). Cells transfected without DNA (Mock) or with a GFP expressing construct (GFP) were included as negative and positive controls, respectively. Molecular weight marker (M; in kDa) is sho [file pone.0225081.s002.tif]
