# Supplementary material for: Complex alternative splicing of human Endonuclease V mRNA, but evidence for only a single protein isoform
Source: PLoS One. 2019 Nov 8;14(11):e0225081. doi: 10.1371/journal.pone.0225081 (PMC6839837; doi:10.1371/journal.pone.0225081)
Supplement: S3 Fig — (PDF) [file pone.0225081.s003.pdf]

Fig. 2B (1st panel)

833K

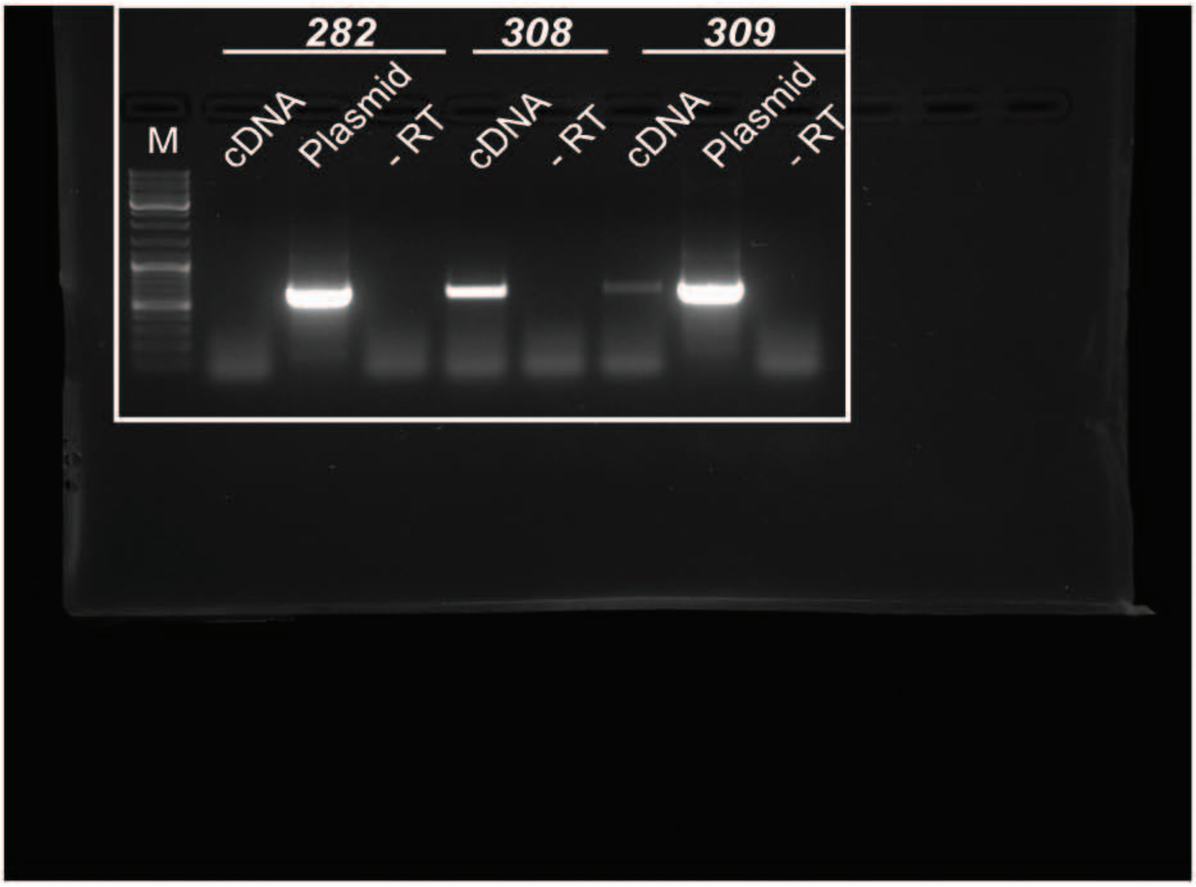

Image capture: UV imaging
Image Lab TM version 5.1 (Bio-Rad)

Fig. 2B (2nd panel)

HAP1

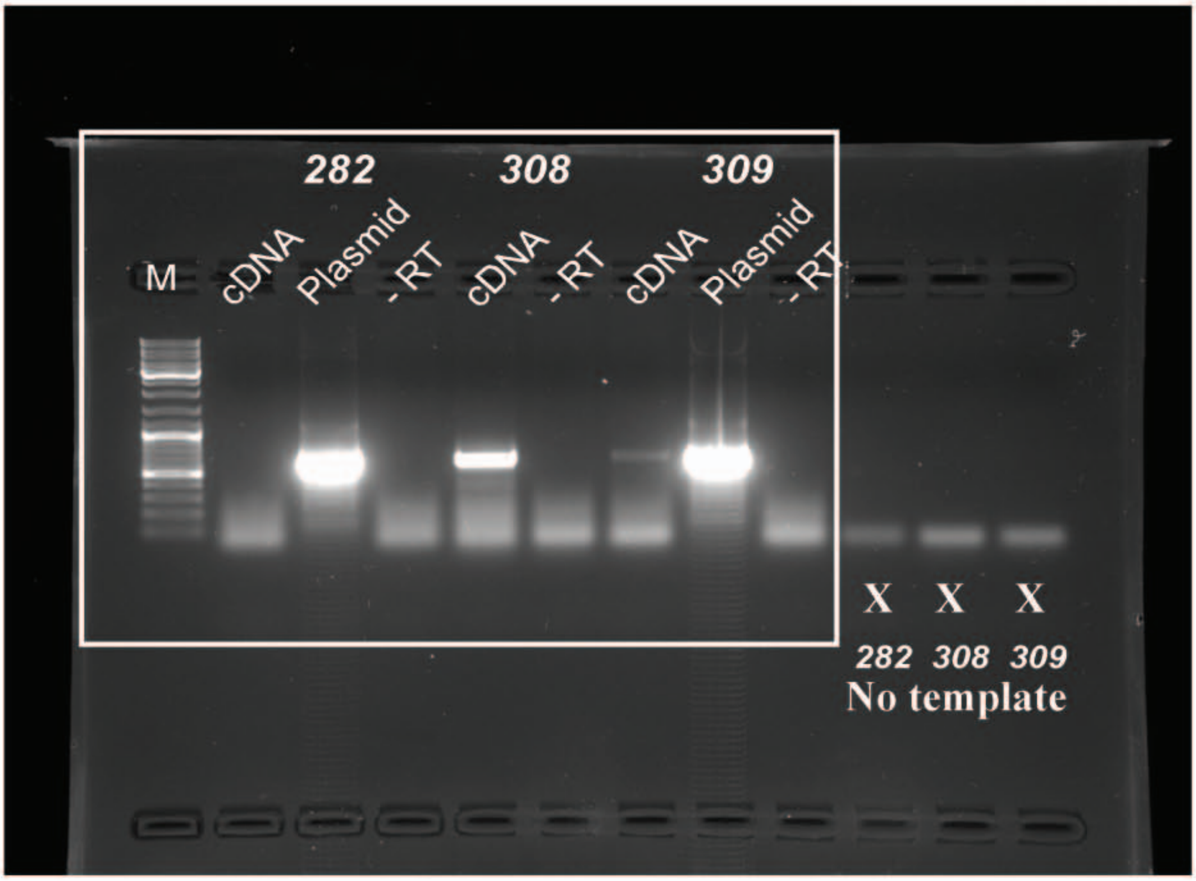

Image capture: UV imaging
Image Lab TM version 5.1 (Bio-Rad)

Fig. 2B (3rd panel)

Huh-7

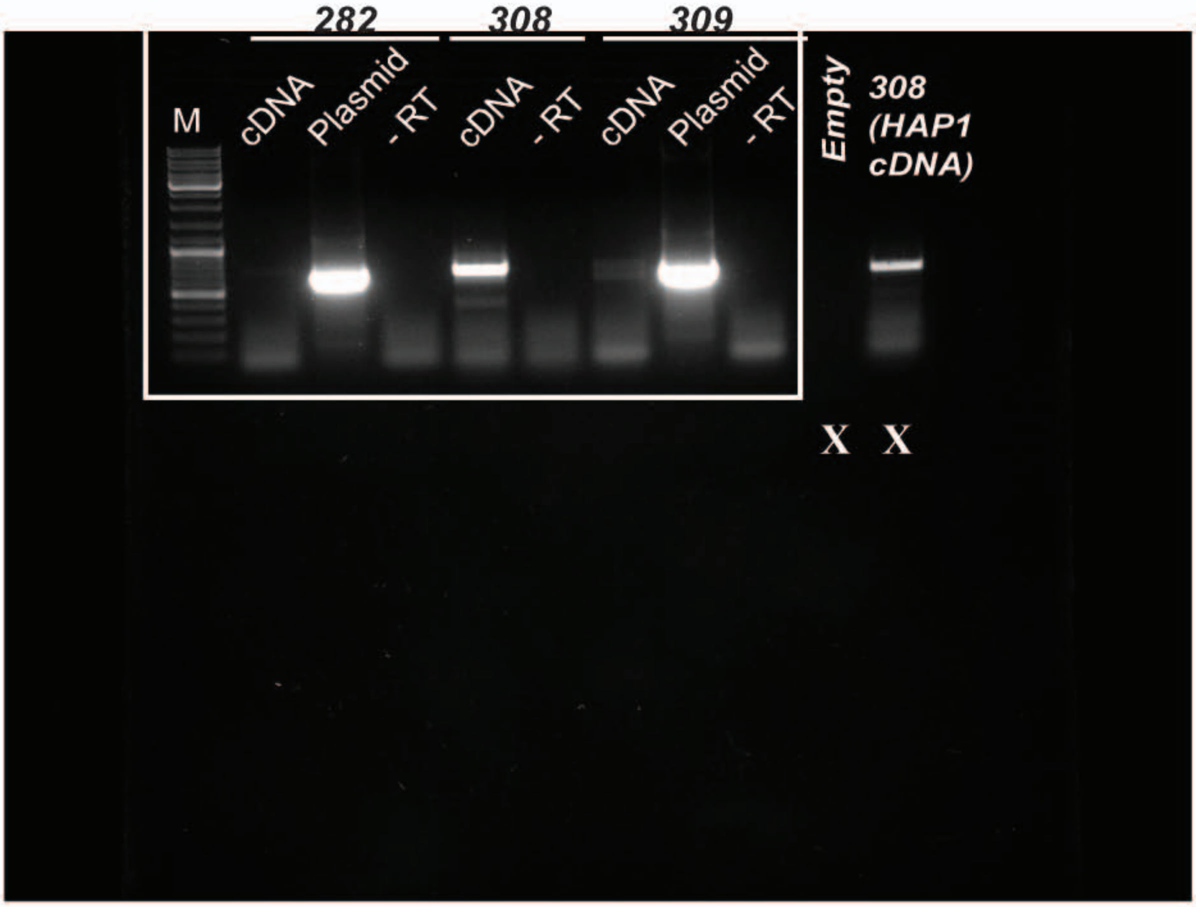

Image capture: UV imaging
Image Lab TM version 5.1 (Bio-Rad)

Fig. 2B (4th panel)

HEK 293T

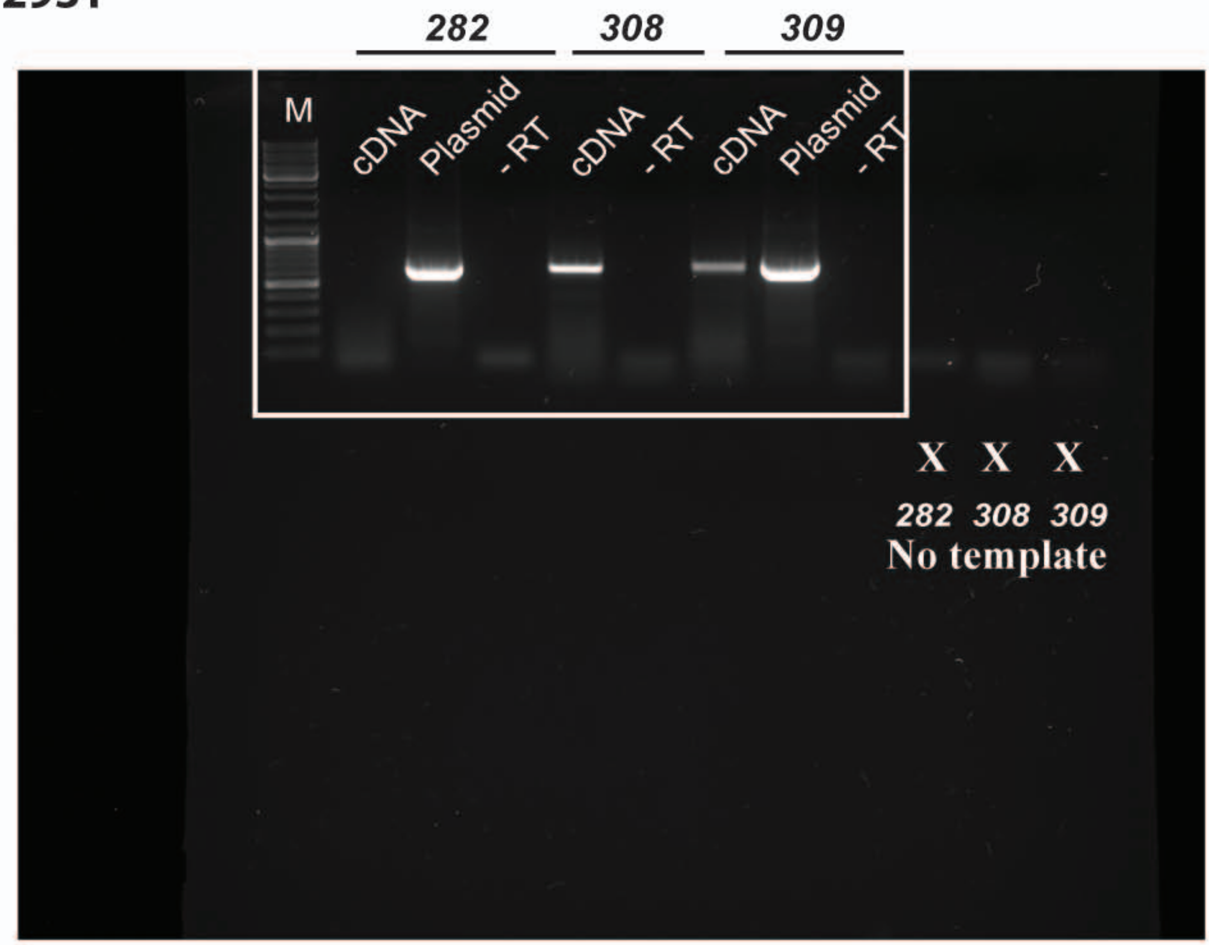

Image capture: UV imaging
Image Lab TM version 5.1 (Bio-Rad)

Fig 3A (upper panel)

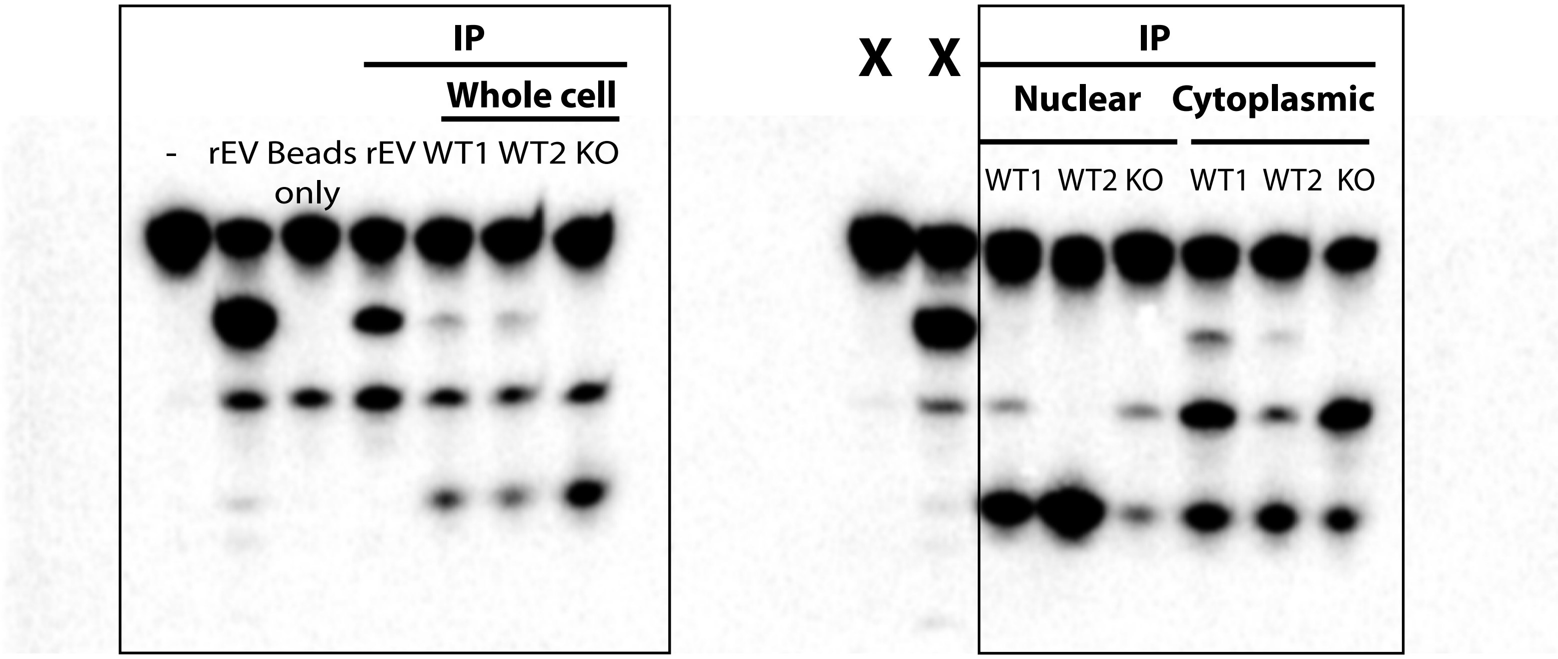

ssIIUI

Image capture: Phosphorimaging

Fig 3A (middle panel)

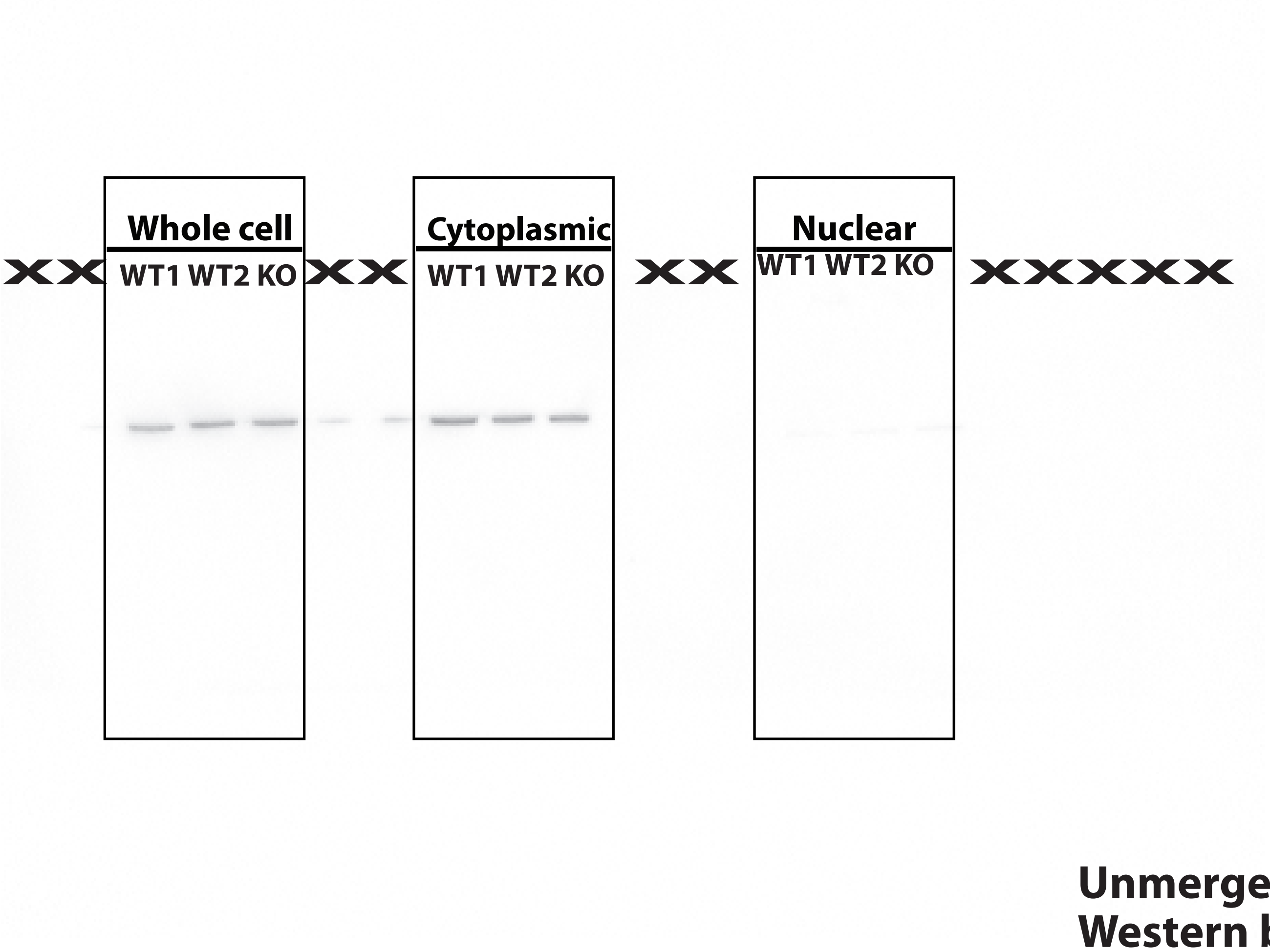

Unmerged
Western blot
Anti-α-Tubulin
Image capture: ChemiLuminescence
Image Lab TM version 5.1 (Bio-Rad)

Fig 3A (bottom panel)

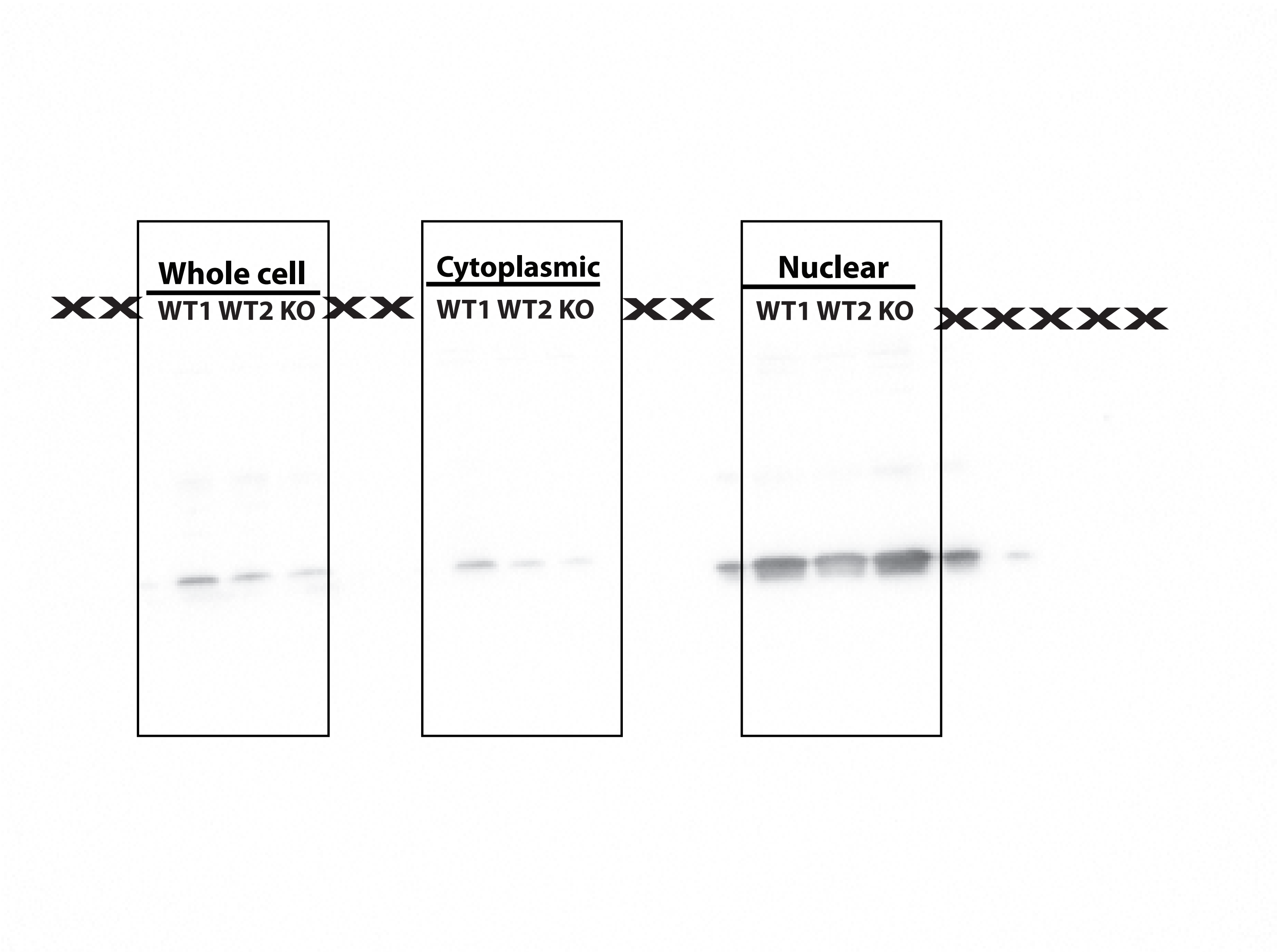

Unmerged Western blot

Anti-Histone H3

Image capture: ChemiLuminescence
Image Lab TM version 5.1 (Bio-Rad)

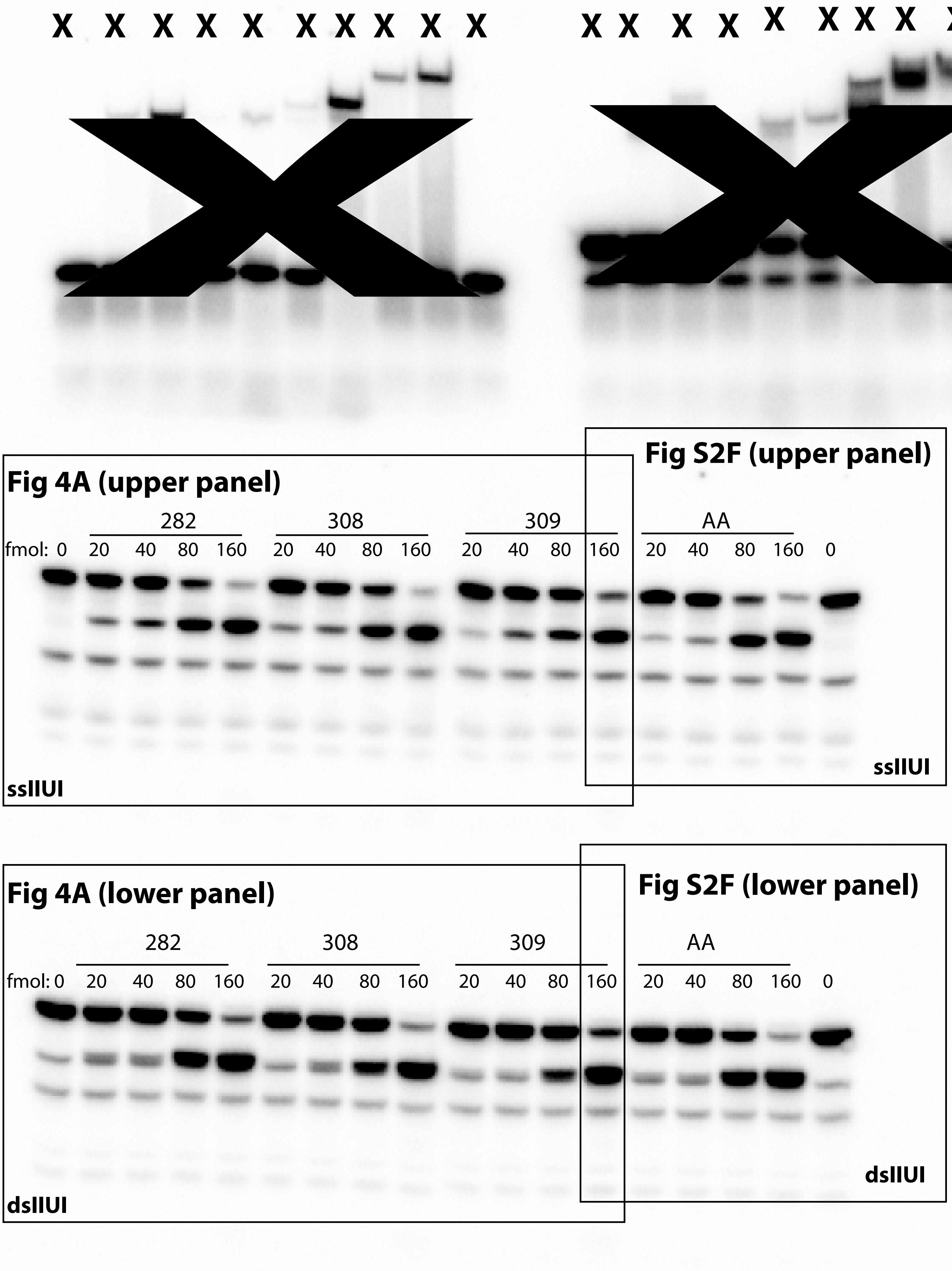

Image capture: Phosphorimaging

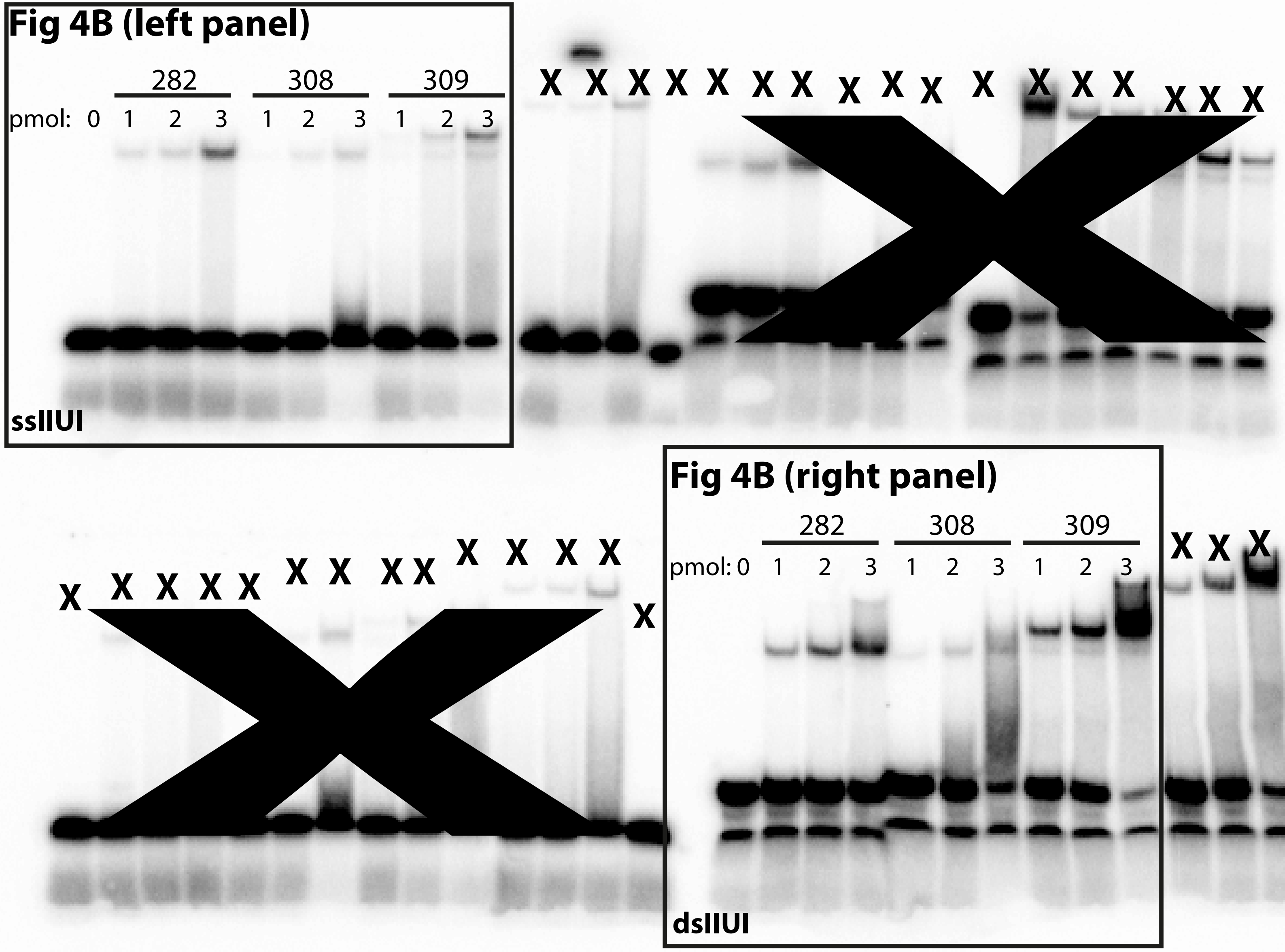

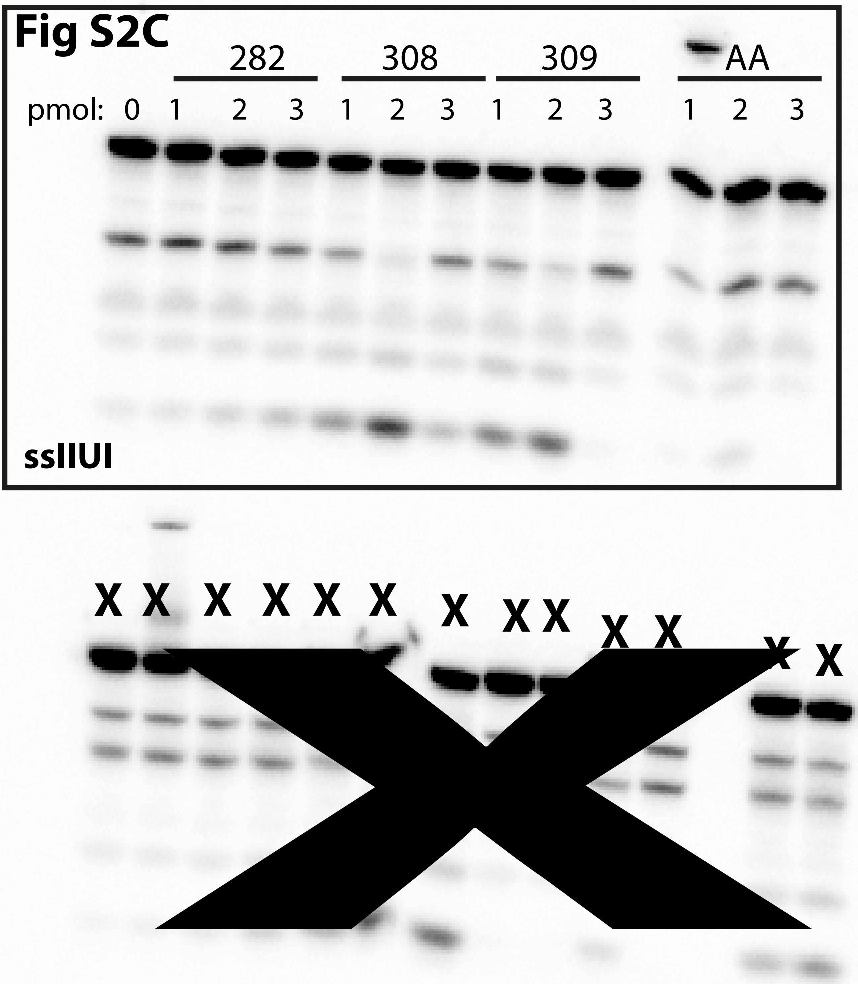

Image capture: Phosphorimaging

Fig4C (1st and 2nd panel)

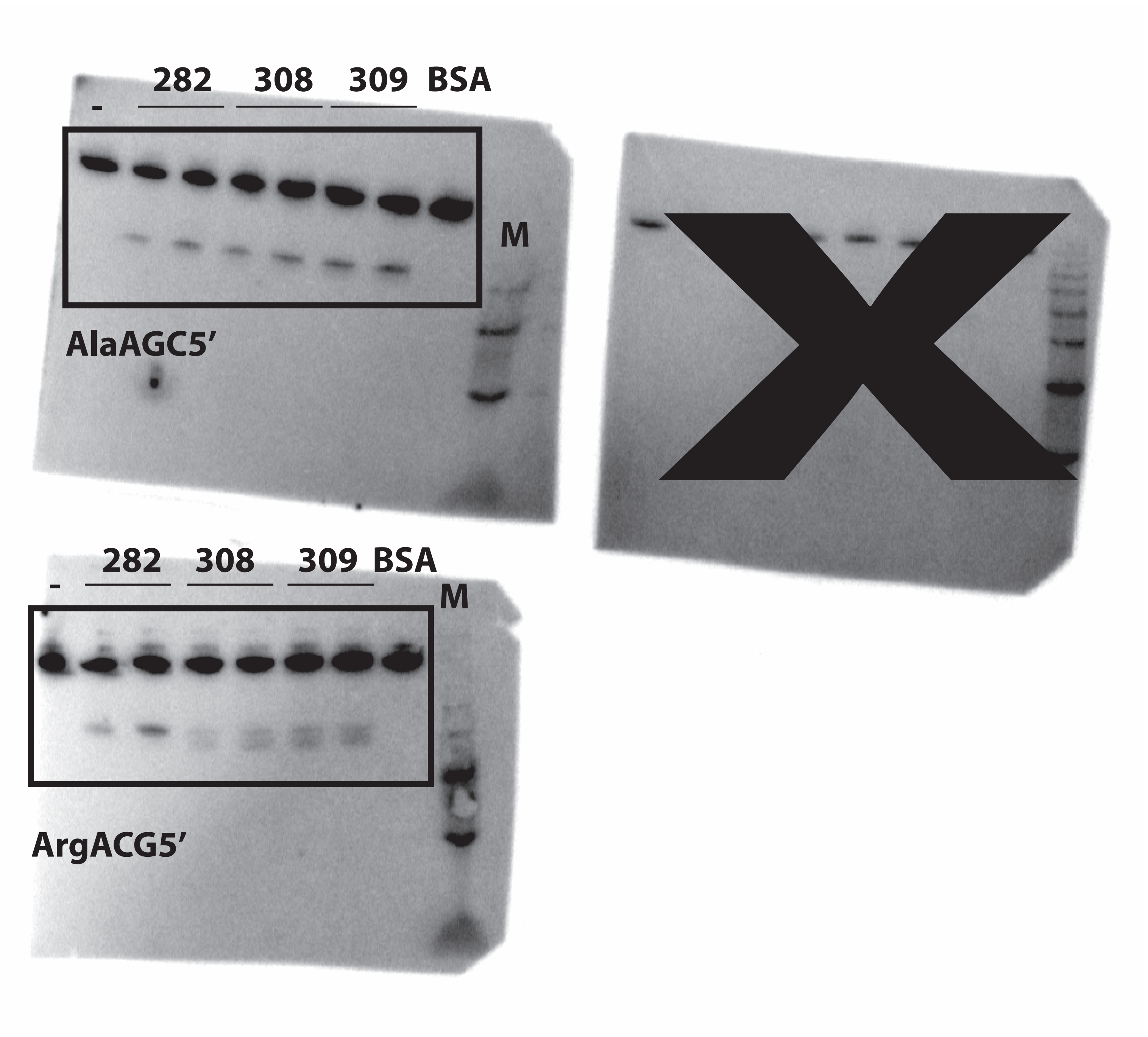

Image capturing: Phosphorimaging

Fig4C (3rd panel)

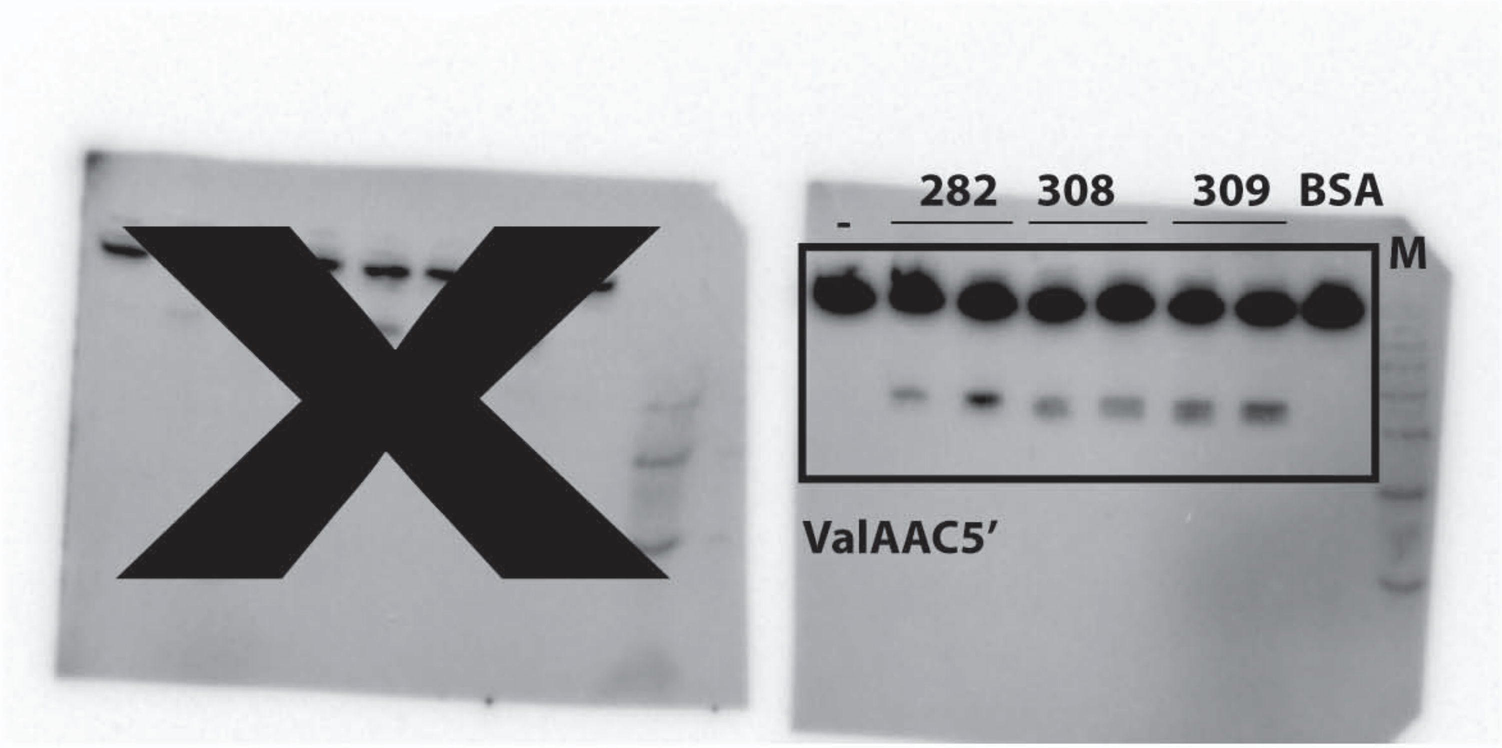

Image capturing: Phosphorimaging

Fig4C (4th panel)

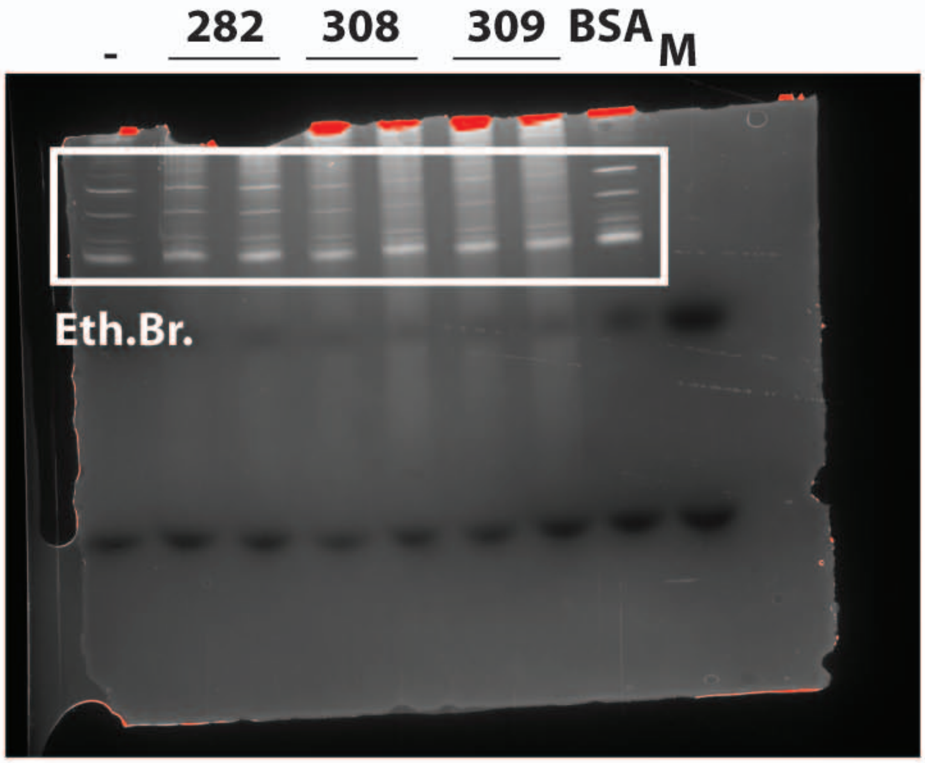

Image capturing: UV imaging
Image Lab TM version 5.1 (Bio-Rad)

Fig S2A

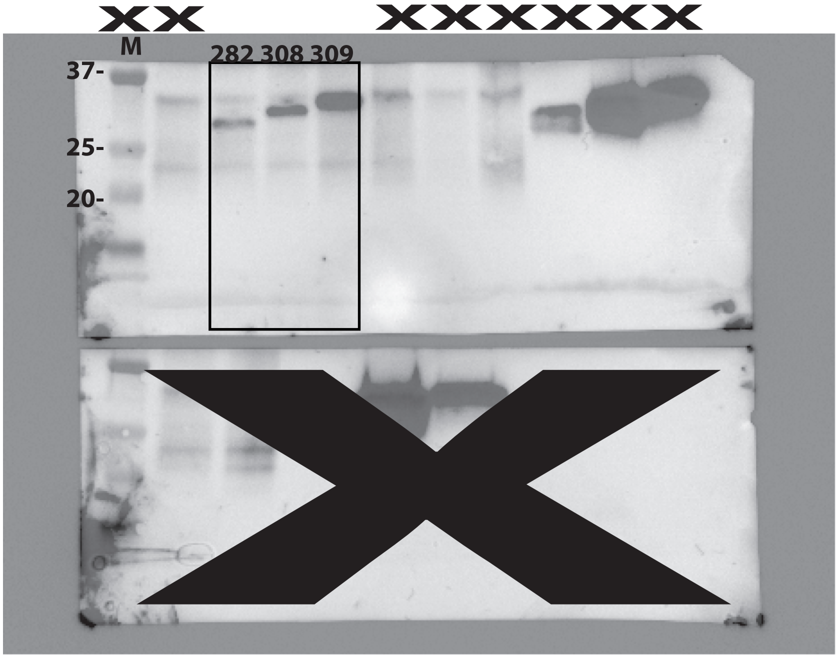

Western blot
Anti-hENDOV

Image capture: ChemiLuminescence
Image Lab TM version 5.1 (Bio-Rad)

Fig S2B

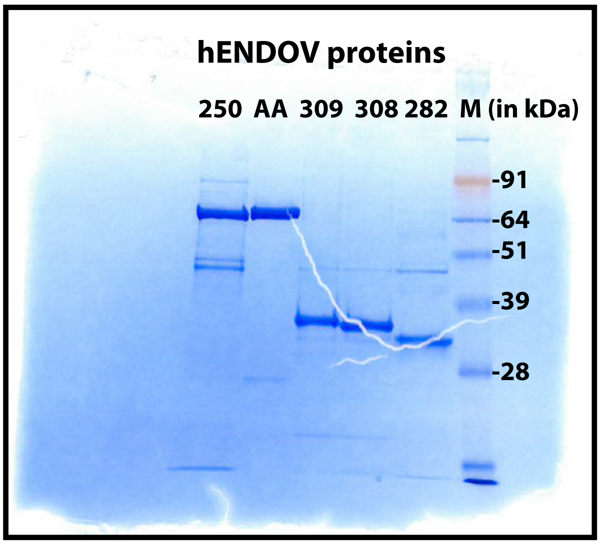

Image capture: Photocopier

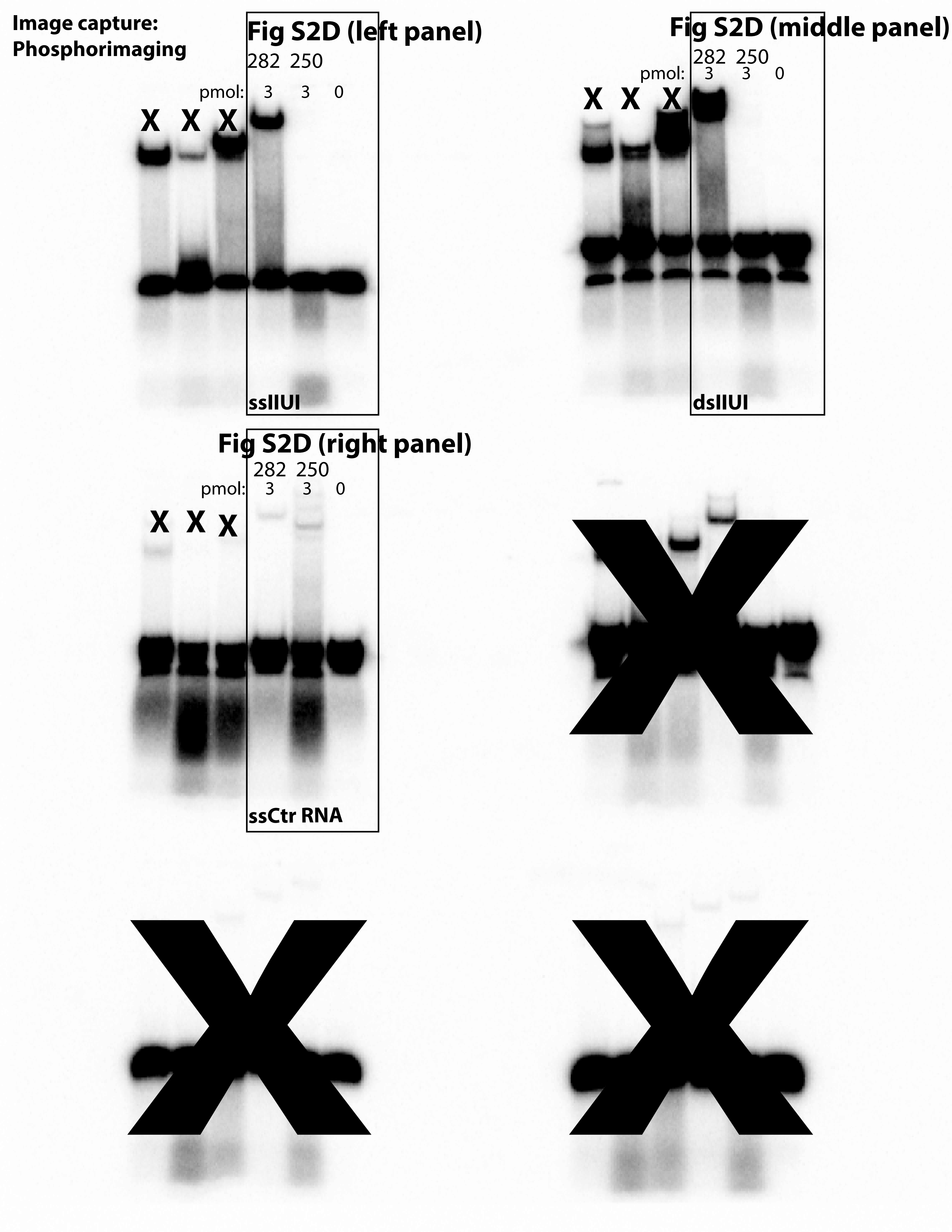
Image capture:
Phosphorimaging
Fig S2D (left panel)
282 250
pmol: 3 3 0
X X X
ssIIUI
Fig S2D (middle panel)
282 250
pmol: 3 3 0
X X X
dsIIUI
Fig S2D (right panel)
282 250
pmol: 3 3 0
X X X
ssCtr RNA

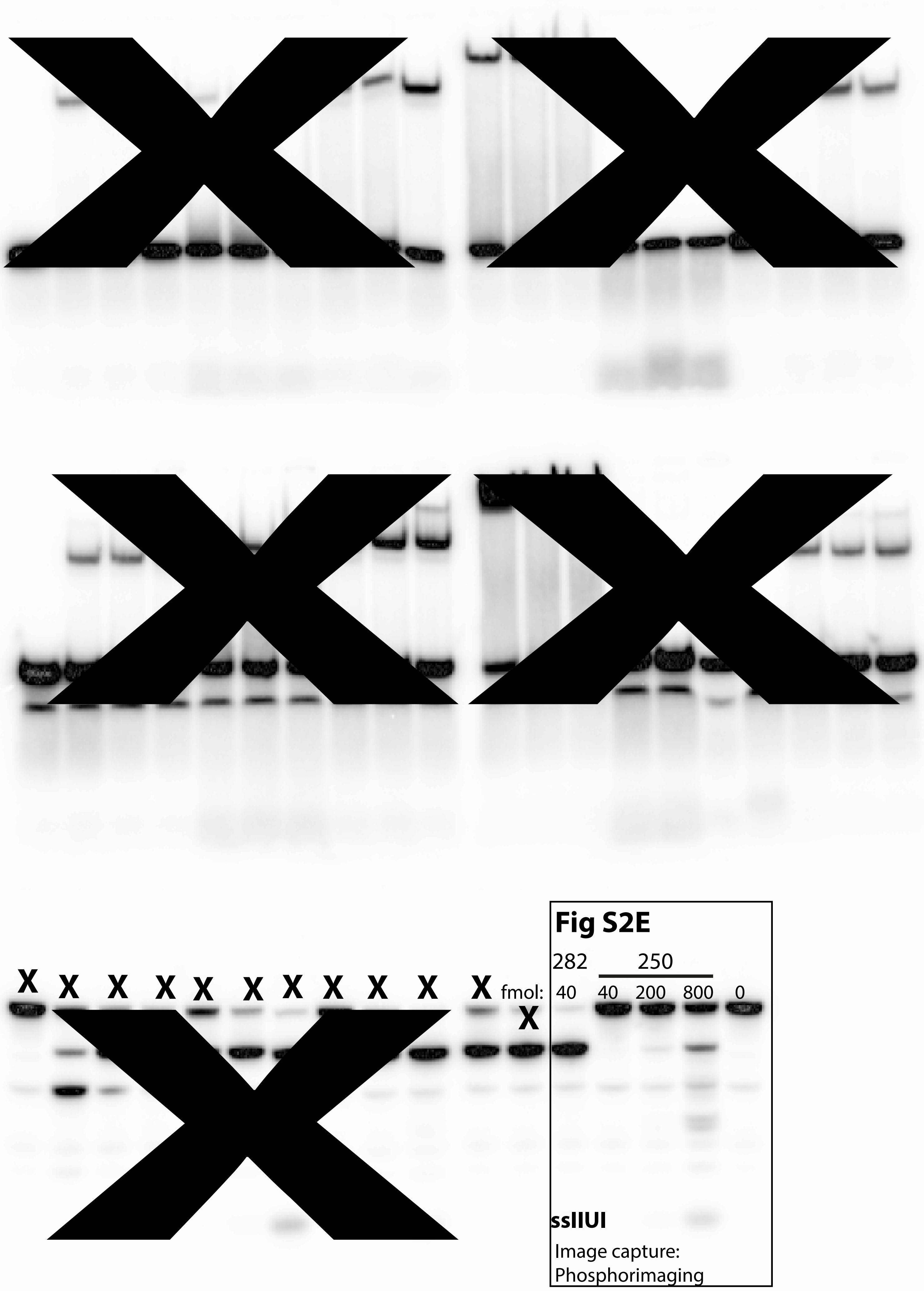
X X X X X X X X X X X
fmol:
X
Fig S2E
282
250
40
40 200 800 0
ssIIUI
Image capture:
Phosphorimaging

Fig S2G (1st panel)

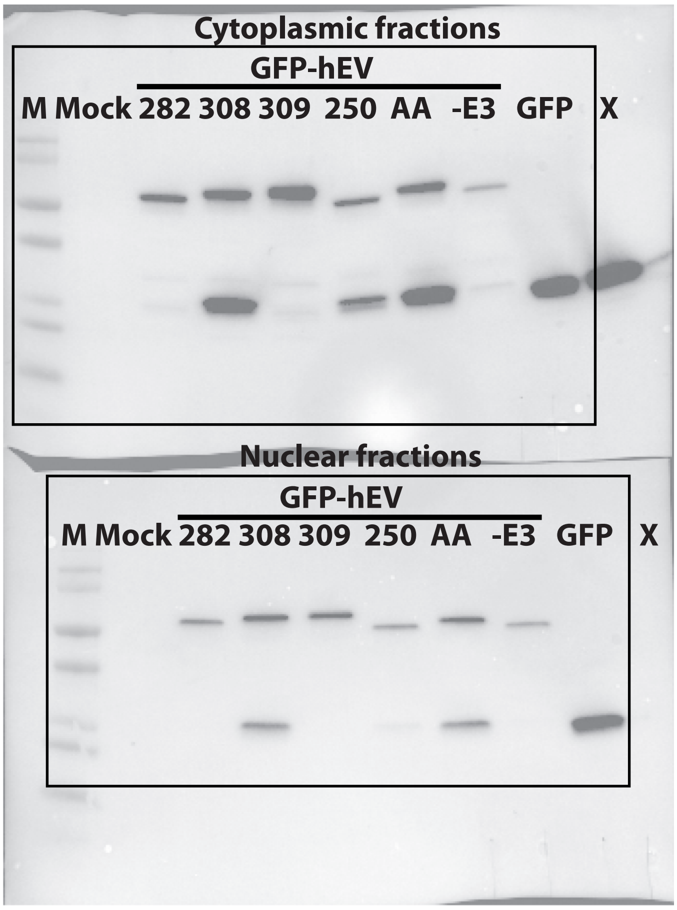

Western blot
Anti-GFP
Image capture: ChemiLuminescence
Image Lab TM version 5.1 (Bio-Rad)

Fig S2G (2nd panel)

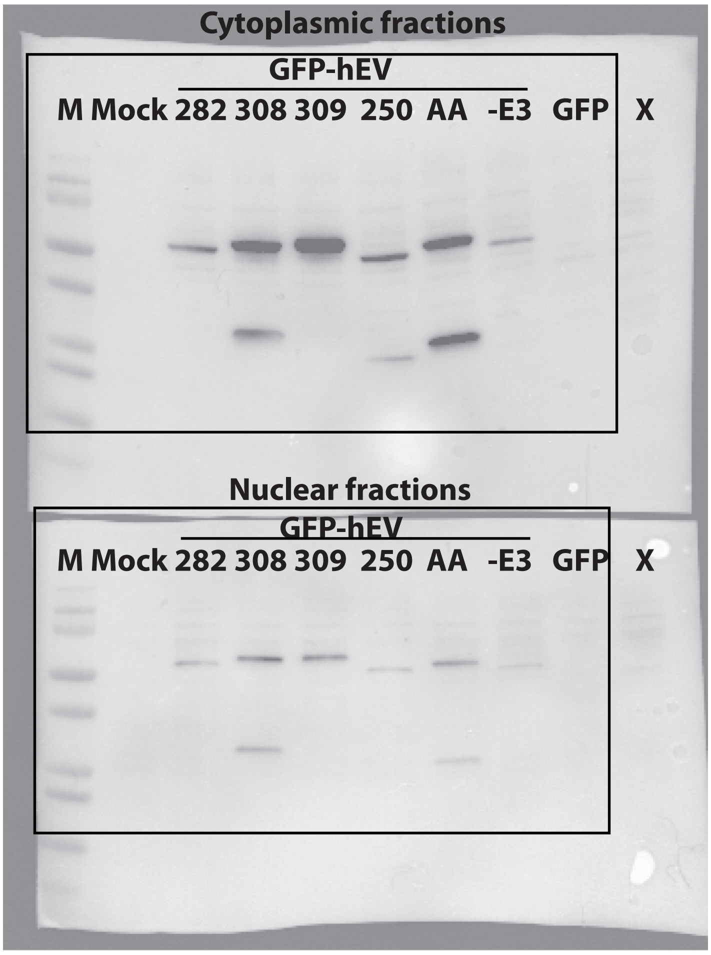

Western blot
Anti-hENDOV
Image capture: ChemiLuminescence
Image Lab TM version 5.1 (Bio-Rad)

Fig S2G (3rd panel)

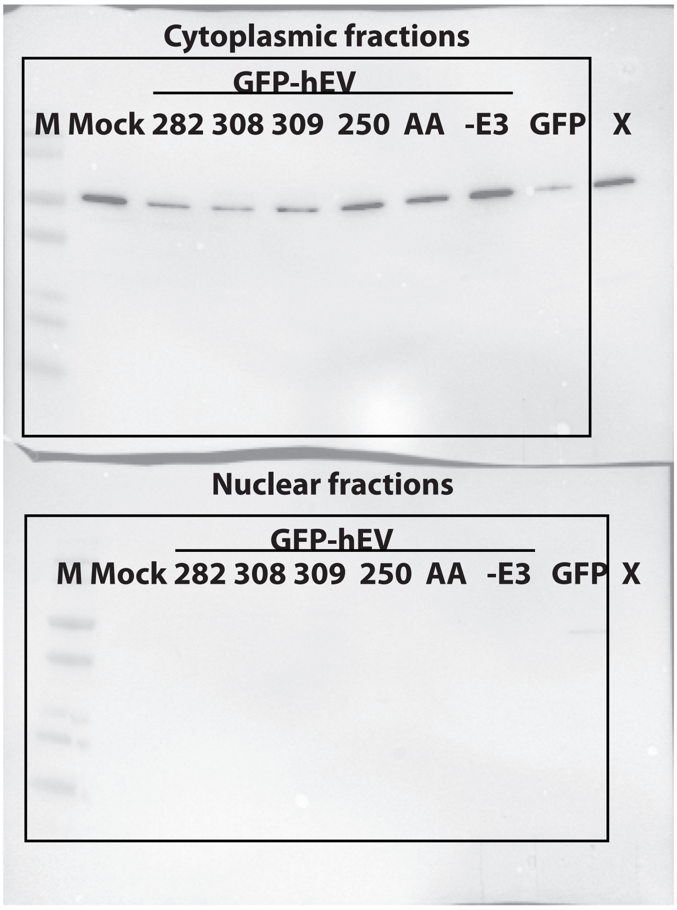

Western blot
Anti-α-Tubulin

Image capture: ChemiLuminescence
Image Lab TM version 5.1 (Bio-Rad)

Fig S2G (4th panel)

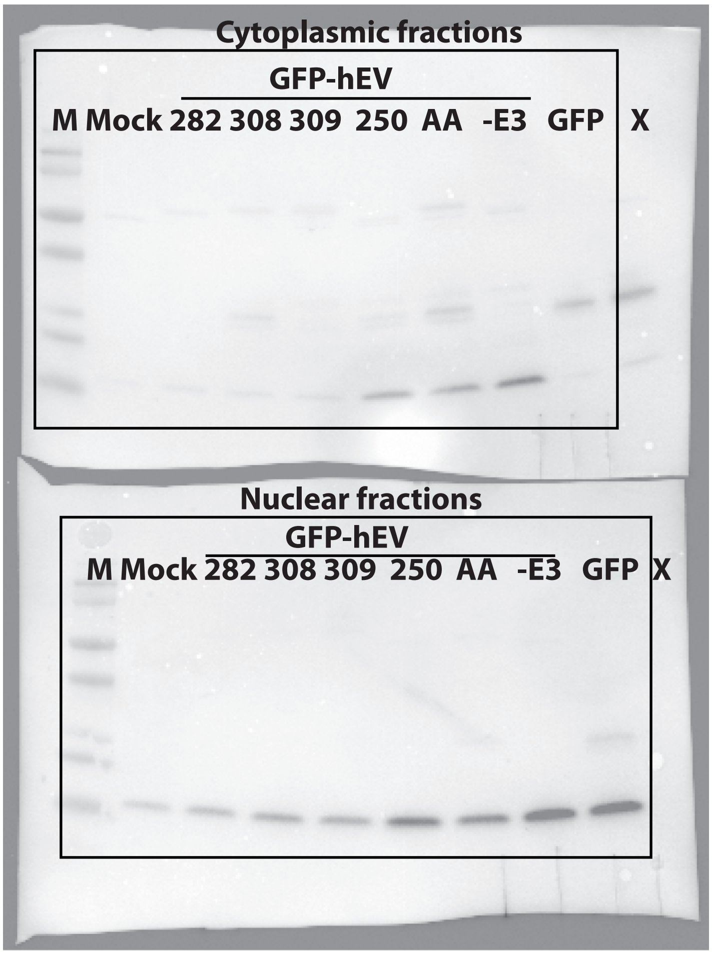

Western blot
Anti-Histone H3
Image capture: ChemiLuminescence
Image Lab TM version 5.1 (Bio-Rad)
